# Ethnobotanical Uses, Phytochemical Composition, Biosynthesis, and Pharmacological Activities of *Carpesium abrotanoides* L. (Asteraceae)

**DOI:** 10.3390/plants11121598

**Published:** 2022-06-17

**Authors:** Sabrin R. M. Ibrahim, Sana A. Fadil, Haifa A. Fadil, Rawan H. Hareeri, Hossam M. Abdallah, Gamal A. Mohamed

**Affiliations:** 1 Department of Chemistry, Preparatory Year Program, Batterjee Medical College, Jeddah 21442, Saudi Arabia; 2 Department of Pharmacognosy, Faculty of Pharmacy, Assiut University, Assiut 71526, Egypt; 3 Department of Natural Products and Alternative Medicine, Faculty of Pharmacy, King Abdulaziz University, Jeddah 21589, Saudi Arabia; safadil@kau.edu.sa (S.A.F.); hmafifi@kau.edu.sa (H.M.A.); gahussein@kau.edu.sa (G.A.M.); 4 Department of Clinical and Hospital Pharmacy, Faculty of Pharmacy, Taibah University, Almadinah Almunawarah 30078, Saudi Arabia; hfadil@taibahu.edu.sa; 5 Department of Pharmacology and Toxicology, Faculty of Pharmacy, King Abdulaziz University, Jeddah 21589, Saudi Arabia; rhhareeri@kau.edu.sa; 6 Department of Pharmacognosy, Faculty of Pharmacy, Cairo University, Cairo 11562, Egypt

**Keywords:** *Carpesium abrotanoides*, Asteraceae, sesquiterpenes, sesquiterpene dimers, bioactivities, biosynthesis

## Abstract

*Carpesium abrotanoides* L. (Asteraceae) is a medicinal plant with immense therapeutic importance and bioactivities. It is commonly encountered in various Asian regions. It has numerous ethnomedicinal uses for curing diverse ailments such as toothache, stomach ulcer, boils, tonsillitis, bronchitis, bacterial infection, bruises, swelling, virus infection, fever, and amygdalitis, as well as an anthelmintic versus round-, tape-, hook-, and pinworms. Different classes of phytoconstituents such as sesquiterpenes, sesquiterpene dimers, monoterpenes, and nitrogenous compounds have been reported from this plant. These phytoconstituents have proved to possess anti-inflammatory, cytotoxic, antimicrobial, and insecticidal capacities. The present review aims to summarize all published data on *C. abrotanoides* including traditional uses, phytoconstituents, bioactivities, and toxicological aspects, as well as the synthesis and biosynthesis of its metabolites through an extensive survey on various databases and various publishers. These reported data could draw the attention of various natural-metabolite-interested researchers and medicinal chemists towards the development of this plant and/or its metabolites into medicine for the prevention and treatment of certain illnesses. Despite the diverse traditional uses of *C. abrotanoides*, there is a need for scientific evidence to support these claims. Clinical trials are also required to further assure these data and validate this plant utilization in treating several diseases.

## 1. Introduction

Medicinal plants have remarkable participation in the separation and discovery of new drugs. Obviously, there is increasing interest in using medicinal plants as part of various traditional medicines for treating various ailments [1,2,3]. The side effects, high cost, and therapeutic limitations of available medications are principal factors that encourage the revivification of herbal treatment [4]. It was also reported that more than 80% of people worldwide rely on traditional medicines as a primary source of healthcare, involving the utilization of plant extracts or their phytoconstituents [4]. Recently, plant-derived metabolites have become greatly important due to their structural diversity and promising bioactivities [5,6,7].

*Carpesium* genus (Asteraceae) comprises about 21 species worldwide [8]. Its species are found in Europe and Asia, particularly in Southwest China mountainous [9]. Its plants are utilized as a traditional remedy in some Asian regions for various complaints such as colds, fevers, insects, bruises, inflammation, and snake bites [9]. Its frequent phytochemically investigated species are *C. cernuum* L., *C. abrotanoides* L., *C. rosulatum* Miq., *C. lipskyi* C.Winkl., *C. triste* Maxim, *C. macrocephalum* Franch. & Sav., *C. rosulatum* Miq., and *C. divaricatum* Sieb. & Zucc.. They have been stated to biosynthesize structurally varied metabolites, including mono-, sesqui-, and di-terpenoids, flavonoids, and phenolics [9]. These metabolites displayed cytotoxicity, anti-parasitic, insecticidal, anti-tumor, anti-inflammatory, antimicrobial, and antioxidant properties [9]. *C. abrotanoides* L. is widely encountered in eastern Asia, Korea, China, Vietnam, Japan, India, Iran, Myanmar, and Russia [10,11]. *C. abrotanoides* has different synonyms; *Amphirhapis wightiana* Hook.f., *C. abrotanoides* var. *thunbergianum* (Siebold & Zucc.) Makino, *C. racemosum* Wall., *C. wulfenianum* Bertol, *C. thunbergianum* Siebold & Zucc., and *C. wulfenianum* Schreb. ex DC. [12]. The plant is a perennial highly branched herb having simple, elliptic, and alternate leaves. It possesses small branched clusters of flower-heads (capitula) that arise from the leaf axils [10]. Each capitulum has yellow 130–300 tiny, densely packed florets that develop into longitudinal and cylindrical ribbed achenes (fruit) (≈1 mm diam. × 3.5 mm long) [10]. *C. abrotanoides* has been used in various traditional medicines for treating various health concerns and had an array of bioactivities including cytotoxic, antiparasitic, insecticidal, antidiabetic, antioxidant, and anti-inflammatory. Furthermore, it was found to yield diverse metabolites such as sesquiterpenes, dimeric sesquiterpenes, monoterpenes, sterols, and aliphatic and nitrogenous compounds with promising bioactivities. Although, the substantial role that *C. abrotanoides* plays in Asian countries’ traditional medicine practice, there is no current review article that deals specifically with this plant. As a result, the present review summarized all published data regarding its phytoconstituents, traditional uses, bioactivities, and toxicity, as well as the reported biosynthesis and synthesis of its metabolites. This review can supply the researchers with knowledge about the recent research developments on *C. abrotanoides* that can help them for quick identification of secondary metabolites and their possible biological activity evaluation. Additionally, we intend to shed light on one of the important medicinal plants and to attract the interest of natural products researchers for more development and utilization of the reported metabolites from it as potential drug leads. Additionally, this work may reflect the existing gaps and highlight the need for *C. abrotanoides’* traditional uses in scientific validation. Moreover, summarizing the reported bioactivities may be significant in identifying the potential therapeutic applications that have not yet been explored for *C. abrotanoides* or its constituents.

## 2. Methodology

To carry out an extensive literature review on *C. abrotanoides*, the data were collected from various scientific databases (e.g., Scopus, Science-Direct, Pub-Med), Publishers (Springer, Taylor & Francis, MDPI, Bentham, Wiley Online Library, and Thieme), and Google Scholar, as well as books and abstracts of articles published in non-English languages. A total of 84 articles were listed in this work.

## 3. Traditional Uses and Clinical Trials

This plant has been utilized in various Asian regions for treating diverse complaints. In Chinese traditional medicine, its fruit (Carpesii Fructus, He Shi, Heshi, Nan-He-Shi, and Bei-He-Shi) have been utilized for treating enterobiasis, ancylastomiasis, cestodiasis, and ascariasis particularly in children, in addition to parasitic infection-associated abdominal pain [13,14]. Additionally, it is very efficient in killing tapeworms and roundworms. In Korea, Japan, and China, its aerial parts were employed orally for various ailments such as toothache, stomach ulcer, boils, tonsillitis, bronchitis, bacterial infection, bruises, swelling, virus infection, fever, and amygdalitis, as well as against snake bites and insects [9,15]. In Southwest China, especially in Miao and Tujia minorities, it is called Tianmingjing and was utilized for bruises, bronchitis, and hepatitis [16]. The herb’s oral decoction is utilized for treating chronic inflammatory disorders [17]. It is called Tenmyoseishi in Japan. In India, it is called “Ban sario” and its root, leaf, and seed infusions are utilized for intestinal worm killing by locals of the Kishtwar plateau [18]. The whole plant was traditionally utilized as an insecticide to control the vegetable insect pests such as *Euxoa segetum* and *Pieris rapae*. Moreover, the whole plants for controlling insect pests in the stored product were placed into the grain depot such as *Tribolium castaneum* and *Sitophilus zeamais* in China [10]. Furthermore, in Korea, *C. abrotanoides* was traditionally consumed as a wild vegetable [19] and the shoots are employed for treating fever and inflammation [20]. A water decoction prepared using *C. abrotanoides*, *Quispualis indica*, and *Areca catechu* (9 g, each) is utilized orally for hook-, round-, pin-, and tapeworm. A handwash (1.5 kg *C. abrotanoides* + 4 kg H_2_O, decoction for 2 h until a volume of 1500 mL, then filtered) was utilized as a skin disinfectant and for handwashing before operation [11].

It was reported that the orally administrated Chinese medicine contains Fructus *Carpesii*, in addition to Semen *Arecae*, Fructus *Ulmus preparatium*, Fructus *Quisqualis meat*, Radix *Glycyrrhizae*, and Fructus *Toosendan* in the form of decoction for two days in the empty stomach caused 89% cure rate in 18 belly worm infected patients [21]. The plant root has been utilized for treating trigeminal neuralgia, oral cavity erosion, folliculitis, herpes zoster, and flat wart in the clinic [9].

## 4. Pharmacological Activities of Crude Extracts

### 4.1. Cytotoxic Activity

Shen et al. stated that the total sesquiterpene lactone and ethanol extracts of the entire plant displayed cytotoxicity capacities versus HepG-2 cells (IC_50_ 4.2 and 28.3 µg/mL, respectively) [16]. Additionally, the aerial part benzene extract was reported to exhibit cytotoxic effectiveness versus HL60 and L1210 (ED_50_ < 5 µg/mL) [22]. The plant EtOH extract was found to induce NQO1(NAD(P)H:quinone oxidoreductase) activity via Nrf2 (NF-E2-related factor 2) nuclear accumulation in HCT116. Interestingly, It had antiproliferative and apoptotic potential as observed by G2/M cell cycle arrest, phosphatidylserine externalization, chromatin condensation, elevated sub-G0/G1 content, PARP (poly(ADPribose) polymerase) cleavage, and p53 upregulation. Therefore, the plant produced chemo-preventive potential via inducing apoptosis and phase II detoxification enzymes [19]. The fruits *n*-BuOH, EtOAc, and aqueous fractions had no influence on the RAW 264.7 murine macrophage cell viability (IC_50_ > 100 µg/mL), whereas the CHCl_3_ (conc. 50 µg/mL, IC_50_ 83.65 µg/mL) and hexane-fraction (Conc. > 6.25 µg/mL, IC_50_ 16.14 µg/mL) displayed cytotoxic potential on these cells [20].

Breast cancer represents one of the most prevalent malignant tumors in women with low survival rates. Chai et al. evaluated the anticancer capacity of root pet. ether fraction (Conc. 50 and 100 µg/mL) versus MDA-MB-231 and MCF-7 using MTT assay. The fraction was found to noticeably suppress proliferation and migration and cause apoptosis in both cells [17]. *C. abrotanoides* exerted its potential via prohibiting glycolysis-linked genes, GLUT1 (glucose transporter-1), LDHA (lactate dehydrogenase A), and HK2 (hexokinase-2) and down-regulating PKM2 (pyruvate kinase M2). PKM2 is closely linked to the angiogenesis, migration, and proliferation of cancer cells, as well as autophagy inhibition [23]. Therefore, the plant was established as PKM2/HIF-1*α* (hypoxia-inducible factor-1α) axis inhibitor, revealing its potential versus breast cancer [17]. Additionally, the cytotoxic capacity of the essential oil on various hepatic cancer cells (Hep3B, HepG2, Huh7, and SMMC-7721) and LO-2 normal liver cell line was investigated by Wang et al. in the MTT assay. The oil prohibited the proliferation of all cancer cells (IC_50_s from 41.28 to 130.36 μg/mL), with little cytotoxicity of L-O2 cells (IC_50_ > 300 µg/mL). Furthermore, it caused apoptotic morphological changes and G2/M and S phases cell cycle arrest in HepG2 cells. Additionally, it reduced Bcl-2/Bax ratio and raised caspase-3 and -9 activation, suggesting the involvement of mitochondria-induced apoptosis in this effect [15].

### 4.2. Antiparasitic Activity

*Paralichthys olivaceus* (Olive flounder) is an important South Korean fish species; however, there are serious industrial losses owing to diseases influencing this fish [24]. Scuticociliatosis is one of the prevalent parasitic diseases caused by *Miamiensis avidus,* resulting in high fish mortality [25]. Additionally, this parasite infects various fish species such as turbot, sharks, adult kingfish, and black rockfish [24]. Hydrogen peroxide and formalin are the approved antiparasitic agents for scuticociliatosis in *P. olivaceus* [26]. However, this treatment is non-effective as they cannot enter the internal organs and formalin over-use is carcinogenic. Therefore, effective and safe natural antiparasitic agents versus *M. avidus* are needed.

The fruit EtOH extract (conc. 50, 70, and 100% *v/v*) demonstrated antiparasitic potential versus *M. avidus* as evident by the induced morphological alterations, including cell shrinkage, reduced motility, and lysis, whereas 100% cell lysis was noticed after 2 h treating with 100% extract [24]. Additionally, the 70% and 100% extracts following concanavalin A pretreatment increased CD8-α, IL-8, IL-1β, TNF-α, UGT2B19, CYP-1A, CYP-1B, and CYP-3A4 expression; however, their expression reduced with 50% extract. Furthermore, they reduced HINAE (hirame natural embryo) cell viability with % between 67 and 80% and IC_50_s from 38.39 to 102.3 mg/L, revealing the less cytotoxic potential of 50% EtOH extract than 70% or 100% extract, while still exhibiting antiparasitic activity against *M. avidus* [24]. Therefore, *C. abrotanoides* had potential as an antiparasitic agent versus *M. avidu,s* supporting its utilization as an anti-parasite in the aquaculture industry [24]. However, the main components accountable for this influence need to be specified and in vivo investigations should be carried out to assess its usage in aquaculture.

*Taenia asiatica* cysticerci was in vitro cultured with *C. abrotanoides* H_2_O decoction (Conc. 20, 40, and 60 mg/mL). It was found that the decoction had a prominent killing capacity on the cysticerci with the highest %mortality (95.0%) at conc. 60 mg/mL. The dead cysticercus had shrunk with degenerated sucker tissue and enlarged scolex [27].

### 4.3. Insecticidal Activity

Plants are a substantial pool for discovering novel and safe insect repellents, especially to protect from blood-sucking insect pests such as bed bugs and mosquitoes. *Spodoptera exigua* Hübner is one of the major pests that occurs in fields, vegetables, and flower crops [28]. The repeated use of chemical pesticides resulted in the development of *S. exigua* resistance to different insecticides, leading to difficulty in controlling this pest [29]. A growing interest has been focused on the discovery of phytochemicals with insecticidal potential [30]. Since *C. abrotanoides* has been traditionally utilized as an insecticide in Chinese and Korean medicines. Feng et al. assessed the insecticidal potential of the fruit EtOH extract versus *S. exigua*. The results revealed that the fruit extract displayed a promising antifeedant potential versus the *S. exigua* larvae with FDI (Feeding-deterrence index) of 48.32% when the larvae fed on treated leaves with 10 mg/mL extract [30].

*Aedes aegypti* (L.) is one of the most threatening pests for humans globally [31]. *Ae. aegypti* was reported to have a potential in the transmission of dengue and zika viruses in humans [32]. Various insecticides such as organophosphate (pirimiphos-methyl and malathion), organochlorines (DDT), carbamate (bendicarb and propoxur), and pyrethroids (lambda cyhalothrin, permethrin, and deltamethrin) were used for controlling *Ae. Aegypti*. [33]. However, their repeated use results in increasing resistance to *Ae. aegypti* versus these insecticides in addition to their hazardous effect on humans that necessitates the discovery of natural alternative agents [34].

In 2022, Haris et al. evaluated the herbs’ essential oil repellent potential versus *Ae. aegypti* adult females utilizing the human bait technique before the scotophase period. *C. abrotanoides* oil (doses 330, 165, and 33 μg/cm^2^) possessed immediate and significant repellency as DEET (N,N-diethyl-meta-toluamide) with maximum repellency periods of 315 and 720 min, respectively, at a dose of 330 μg/cm^2^. Furthermore, the repellent potential of caryophyllene (**111**) and *trans*-nerolidol (**112**) (doses 33, 165, and 330 μg/cm^2^), the major constituents of essential oil, was assessed versus *Ae. aegypti*. Interestingly, *Ae. aegypti* landing was completely prohibited for 45 min by **112** when tested at 330 μg/cm^2^, while **111** did not completely prohibit *Ae. aegypti* landing. On the other side, **111**/**112** mixture (dose of 330 μg/cm^2^) completely prohibited *Ae. egypti* landing for 60 min. Therefore, *C. abrotanoides* or *trans*-nerolidol could be formulated as safer, commercial mosquito repellent products [32].

### 4.4. Antidiabetic Activity

Diabetes is a chronic worldwide disease that influences millions of people [35]. It was estimated to be the ninth reason for death with 1.5 million deaths in 2019 [35]. One of its therapeutic approaches for treatment is the carbohydrate absorption suppression after food intake which is aided by the prohibition of α-glucosidase and α-amylase enzymes [36,37]. The main goal of its treatment is to keep the blood glucose levels near normal in both the postprandial and fasting conditions [38]. The aerial parts’ 80% MeOH extract were found to contain remarkable amounts of total phenolics (88 mg gallic acid/g extract) and flavonoids (2 mg quercetin/g extract). It also revealed non-competitive α-glucosidase inhibitory effectiveness (IC_50_ 44.22 µg/mL), compared to acarbose (IC_50_ 2.5 µg/mL) [39].

### 4.5. Antioxidant Activity

The aerial parts’ 80% MeOH extract demonstrated antioxidant potential in various assays: ABTS (2,2′-azinobis-3-ethylbenzothiazoline-6-sulfonic acid), DPPH (1,1-diphenyl-2-picrylhydrazyl), NOS (nitric oxide scavenging), and FIC (ferrous ion chelating) (IC_50_s 15.6, 111.2, 798.5, and 150.2 µg/mL, respectively) compared to ascorbic acid (IC_50_s 6.99 µg/mL for ABTS, 7.55 µg/mL for DPPH, and 220 µg/mL for NOS) and citric acid (IC_50_ 9764 µg/mL for FIC). It also prohibited lipid peroxidation. This influence was attributed to its high phenolic contents [39]. The fruit hexane-fraction (IC_50_ 12.95 mg/mL) was found to have more powerful antioxidant capacity than *n*-BuOH and EtOAc-fractions (IC_50_s 31.17 and 21.43 mg/mL, respectively) in the DPPH method [20]. The 80% EtOH extract of the aerial parts displayed scavenging potential in the DPPH and ABTS assays, as well as reducing power such as butylated hydroxyanisole. It significantly repressed RBC oxidative hemolysis and AAPH (2,2′-azobis (2-methylpropionamidine) dihydrochloride)-induced MDA (malondialdehyde) formation, and GSH depletion in RBCs, indicating its anti-hemolytic potential [40].

### 4.6. Anti-Inflammatory Activity

Jeong et al. assessed the influence of the aerial parts’ EtOH extract on the COX-2 (cyclooxygenase-2) expression by various TLRs (Toll-like receptors) agonists in murine macrophages. TLRs and their signaling components could be a potential therapeutic target for chronic inflammation disorders. *C. abrotanoides* repressed COX-2 expression caused by LPS (lipopolysaccharide, TLR4 agonist), macrophage-activating lipopeptide 2-kDa (TLR6 and TLR2 agonist), and polyriboinosinic polyribocytidylic acid (TLR3 agonist). This indicated its capacity to regulate the TLRs TRIF (Toll-interleukin-1 receptor domain-containing adapter inducing interferon-β)- and MyD-88 (myeloid-differential-factor-88)-dependent pathways, resulting in reduced expression of inflammatory genes; therefore, it could be beneficial for treating chronic inflammatory disorders [41]. In another study, Lee et al. also revealed that the 70% EtOH extract of aerial parts suppressed iNOS (inducible nitric oxide synthase) expression boosted by these TLRs agonists in murine macrophages [42]. Additionally, the blooms’ MeOH extract (dose 10 mg/kg BW) exhibited anti-inflammatory capacity by reducing the elevated IL-6 and IFNγ levels induced by LPS in mice. It also attenuated IL-6, IFNγ, and IL-4 levels in a Con-A inflammation model [43].

The anti-inflammation capacity of various fruit fractions was assessed by Lee et al. [20]. The CHCl_3_ (89.1%, IC_50_ 37.1 µg/mL) and EtOAc (74.4%, IC_50_ 78.35 µg/mL) fractions remarkably prohibited NO production in macrophage cells caused by LPS via NO chemical scavenging and iNOS transcription repression. Moreover, all fractions potently prohibited PGE2 biosynthesis, whereas CHCl_3_ (Conc. 50 µg/mL, 94.5%) and hexane (conc. 6.25 µg/mL, 89.1%) had more potent effectiveness than celecoxib (Conc. 20 µg/mL, 87.3%). This effect was due to the attenuation of LPS-boosted COX-2 expression. Additionally, the EtOAc, hexane, and aqueous fractions repressed IL-1β levels significantly almost to the basal (IC_50_s 5.13, 0.26, and 3.53 µg/mL and %inhibition 77.5–92.8%) [20]. Hence, the plant could have a cartilage protective influence and retard the inflammation process in rheumatoid arthritis [20].

## 5. Phytochemicals and Their Pharmacological Activities

**Figure 1:**
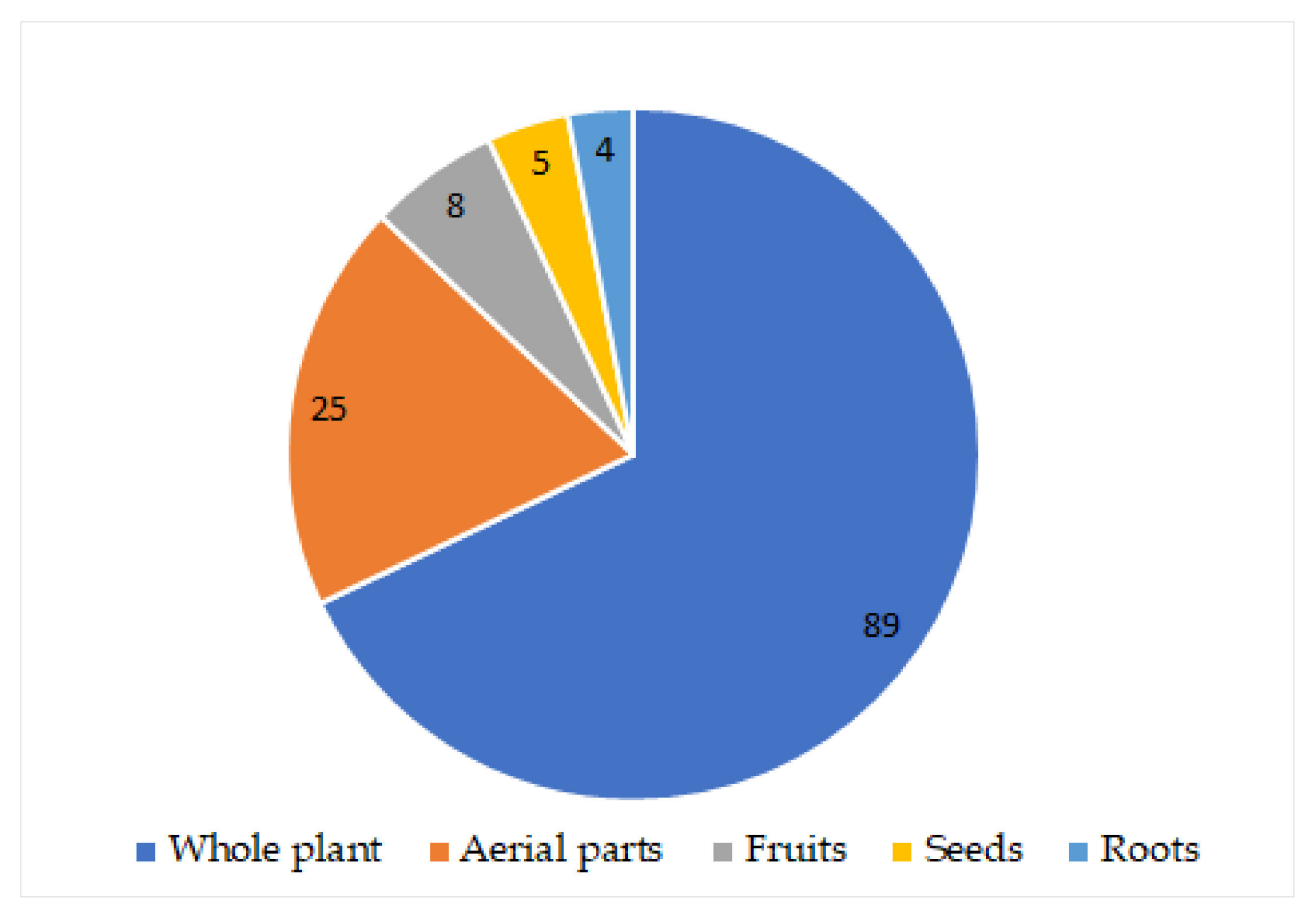
Number of metabolites reported from different parts of *C. abrotanoides*. **89** Compounds from whole plant; 25 compounds from aerial parts; 8 compounds from fruits; 5 compounds from seeds; 4 compounds from roots.

In total, 118 metabolites have been separated from various parts of *C. abrotanoides* using various chromatographic tools, including various terpenoids, sterols, and aliphatic and nitrogenous compounds (Figure 1).

**Figure 2:**
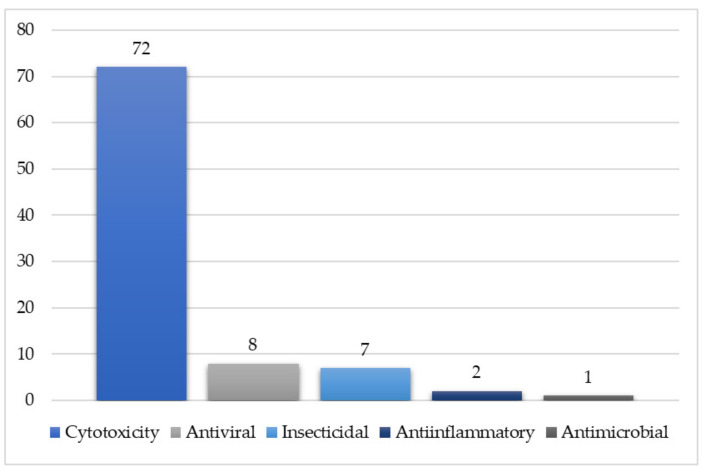
Tested metabolites for each biological activity.

Additionally, some volatile compounds have been characterized from the plant essential oil by GCMS. These metabolites have been assessed for diverse bioactivities (Figure 2). It is obvious that most of the reported metabolites have been evaluated for their cytotoxic potential.

Furthermore, the reported compounds along with their molecular weights/formulae, part of the plant/fraction from which they were isolated, and the place of location are listed in Table 1.

### 5.1. Monoterpenes

**Figure 3:**
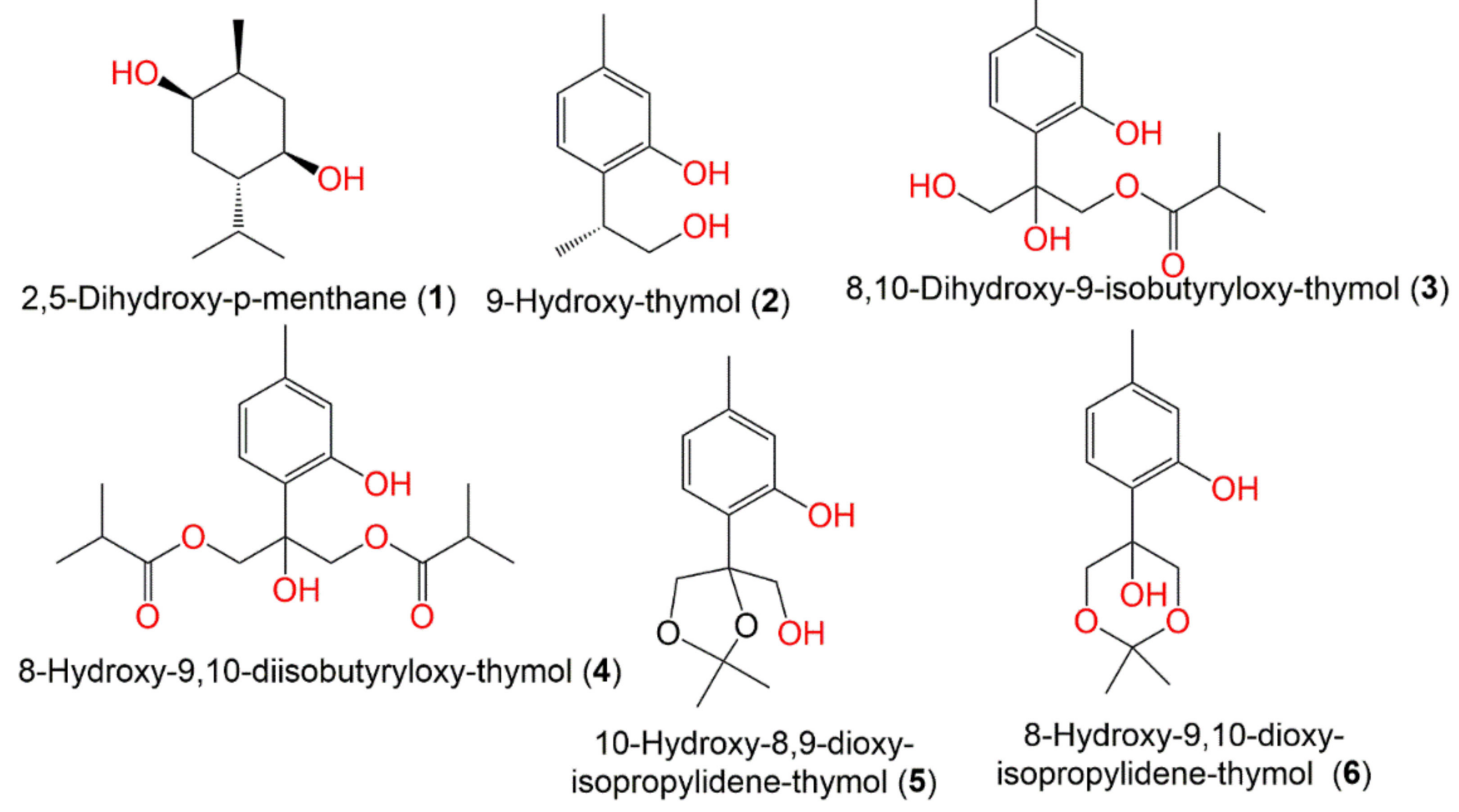
Structures of monoterpenes (**1**–**6**) reported from *C. abrotanoides*.

In 2021, Bao-jia et al. separated thymol-related metabolites from the petroleum ether extract of the whole plant: 8-hydroxy-9,10-diisobutyryloxy-thymol (**3**), 8,10-dihydroxy-9-isobutyryloxy-thymol (**4**), 9-hydroxy-thymol (**2**), 10-hydroxy-8,9-dioxy-isopropylidene-thymol (**5**), and 8-hydroxy-9,10-dioxy-isopropylidene-thymol (**6**) were elucidated through the comparison of their spectral data with the literature. These compounds were assessed for their cytotoxicity (Conc. 40 μM) versus HL60, SMMC7721, A549, SW480, and MCF7 cell lines. Compound **4** revealed a remarkable potential versus MCF-7 and SW480 (%inhibition 72 and 81%, respectively) [45]. In 2016, from the *C. abrotanoides* fruit MeOH extract, Wu et al. separated and characterized a monoterpene, 2,5-dihydroxy-*p*-menthane (**1**) using Sephadex LH-20 and SiO_2_ CC and spectral analyses, respectively (Figure 3). Compound **1** had antifeedant effectiveness versus *Plutella xylostella* third-instar larvae (EC_50_ 43.99 mg/L). Furthermore, it displayed stomach–contact combination toxicity versus *B. odoriphaga* fourth-instar larvae (LD_50_ 68.47 mg/L) [44].

### 5.2. Sesquiterpene Lactones (STLs)

#### STLs Isolation and Identification

Various sesquiterpenes including guaianolide, pseudo-guaianolide, eudesmanolide, germacranolide, eremophilanoide, and STLs with cyclopropane ring in addition to dimeric sesquiterpenes were separated from *C. abrotanoides* using various chromatographic tools. For isolation of STLs, the aerial parts, blooms, fruits, or whole plant was extracted using various solvents (e.g., MeOH, 95% MeOH, 95% EtOH, 70% EtOH) [10,43,68]. The EtOAc or CHCl_3_ fraction of the different extracts was chromatographed using SiO_2_ CH_2_Cl_2_/MeOH, pertroleum ether/EtOAc, petroleum ether/acetone, petroleum ether/CHCl_3_, CHCl_3_/MeOH, petroleum ether/EtOAc/MeOH, cyclohexane/EtOAc), Sephadex LH-20 (CHCl_3_/MeOH 2:3, hexane/CHCl_3_/MeOH 1:2:3, MeOH, MeOH:CH_2_Cl_2_ 1:1, MeOH:CHCl_3_ 1:1), MCI gel CHP20P (EtOH/H_2_O or MeOH/H_2_O gradient), and RP-18 (MeOH/H_2_O gradient) CC [16,54,60,62]. Purification of the isolated STLs was achieved using HPLC (CH_3_CN/H_2_O or MeOH-H_2_O gradient), preparative-TLC (CHCl_3_/acetone 30:1, n-hexane/acetone 5:1, n-hexane/EtOAc/acetone 3:1:1), or HPLC-ESI-QTOF-MS/MS combined with HSCCC (high-speed counter-current chromatography, n-hexane/EtOAc/MeOH/H_2_O, 1:1:1:1, 1:9:9:1, 3:1:1:1 *v/v*/*v/v*), n-hexane/ MeOH/H_2_O (5:4:1, *v/v/v*), and CHCl_3_/MeOH/H_2_O (4:3:2, *v/v/v*) strategy [16,54,60,62]. Their structures and stereo-configurations were verified by various spectral tools (e.g., IR, NMR, HRMS), as well as ECD, X ray, and TDDFT-ECD (time-dependent density-functional-theory electronic circular dichroism) calculations [51,54,63]. These metabolites and their activities are highlighted in this work.

The sesquiterpene lactones, **14**, **28**, **29**, **35**, **40**, **49**, **55**, and **56** were assessed for their cytotoxic effectiveness versus A549, L1210, SK-MEL-2, SK-OV-3, HCT-15, and XF-498 cell lines. They demonstrated (ED_50_ < 20 μM) notable cytotoxic potential versus all cell lines, whereas **14**, **29**, **35**, and **40** displayed potent effectiveness (ED_50_ < 10 μM) compared to cisplatin. It was established that γ-lactone-α-methylene moiety was substantial for activity and the ketone or epoxide group in the cyclopentane enhanced the activity, while oxidation in the cycloheptane reduced activity [68] (Table 2).

**Scheme 1:**
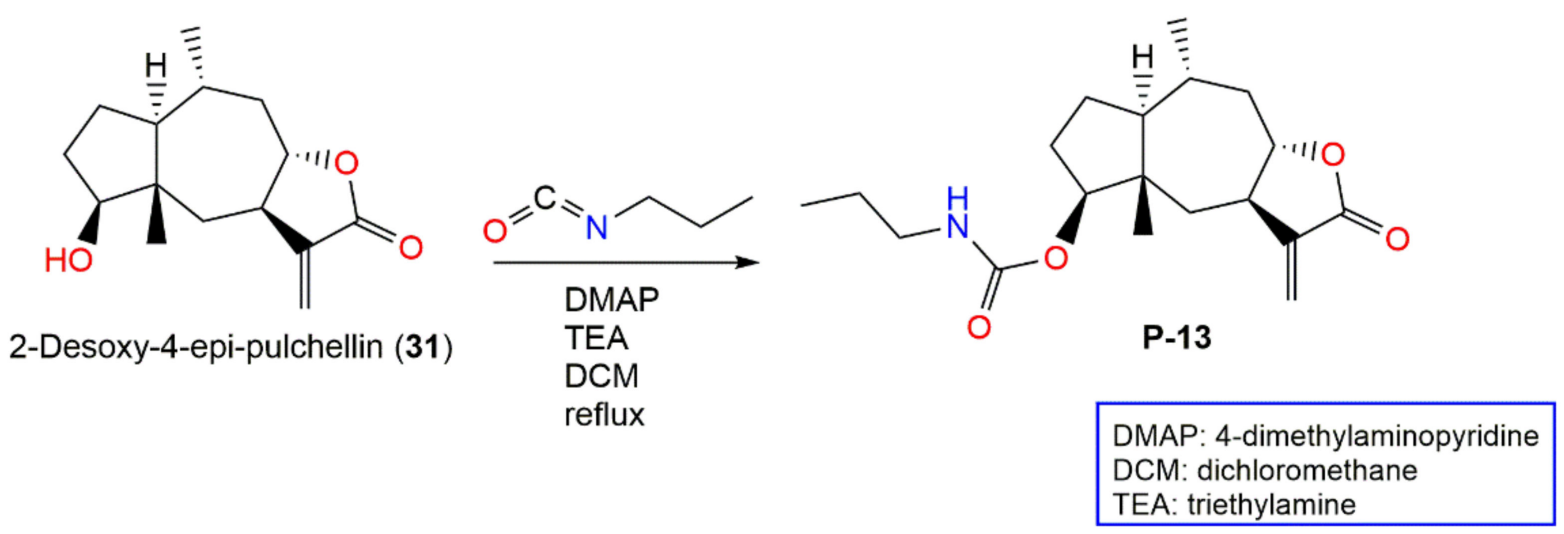
Synthetic pathway of **P-13** [72].

In 2019, Huang et al. synthesized **P-13** (2-desoxy-4β-propylcarbamate-pulchellin), a 2-desoxy-4-epi-pulchellin (**31**) derivative [72] (Scheme 1). This compound (IC_50_ 1.5 μM) had 10 times more potent STAT3 signaling inhibitory potential than **31** in the STAT3-responsive luciferase-gene-reporter assay. P-13 suppressed IL-6-caused STAT3 activation. Additionally, it prohibited JAK2 phosphorylation and JAK2 kinase activity that was blocked by reducing agents, DTT (dithiothreitol) or GSH (glutathione), indicating its thiol-reactive α-β-unsaturated carbonyl functionality involvement. Further molecular docking displayed its covalent binding with the C452 (cysteine-452) in the JAK2 SH2 domain. It also suppressed growth and produced apoptosis on various cancer cell lines, especially those with activated STAT3 and prohibited in vivo tumor growth in mice bearing the HT29 xenografts. Therefore, this derivative represented a novel JAK2-covalent inhibitor and provided a promising candidate for anticancer drugs [72].

**Figure 4:**
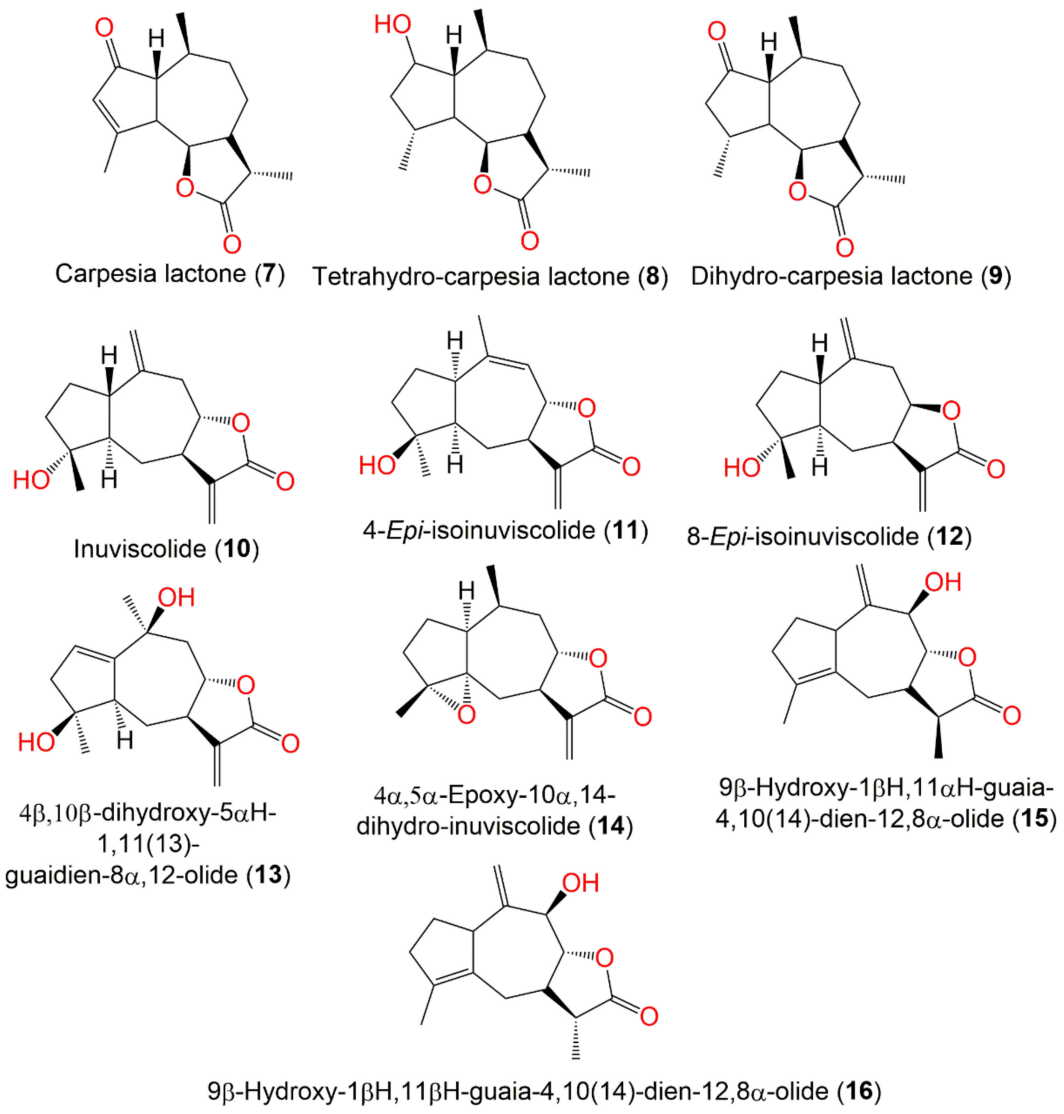
Guaianolide sesquiterpenes (**7**–**16**) reported from *C. abrotanoides*.

Tian et al. investigated the cytotoxic potential of **11** (Figure 4) versus different cancer cells, MDA-MB-231, CaCo-2, A549, CNE-2, SH-SY5Y, HepG-2, and HGC-27, and normal cell lines, Marc-145, GES-1, and MDCK, in the MTT assay. It was found that the MDA-MB-231 cell was the most affected cell (IC_50_ 4.07 µM), whereas **11** had a lower effect on the normal cells, revealing its selectivity. It induced apoptosis and pro-death autophagy in MDA-MB-231 cells through elevating Bax, LC3-II, Bad, and p-ULK1 expression, as well as down-regulation of p-Akt, p-PI3K, Bcl-2, p-mTOR, LC3-I, p62, and Bcl-xl. Hence, it produced its effect through the Akt/PI3K/ mTOR signaling pathway [73].

4α,5α-Epoxy-10α,14-dihydro-inuviscolide (**14**) was separated from the blooms’ MeOH extract [43]. This compound noticeably lessened IL-6, -4, -10, and -13, IFNγ without affecting TNFα in LPS-treated RAW264.7 cells and PMA-treated NK-YS and EL-4 cells. Furthermore, it prohibited PMA-induced IL-10 promoter activity and NF-κB activation as Bay11. It is noteworthy that this compound did not influence PMA-mediated IκB and MAPK kinase phosphorylation, IκB degradation, and NF-κB-p65 nuclear translocation [43]. Therefore, it blocked the cytokine production accompanied with the inflammation by suppressing NF-κB activation. It could be potentially utilized as an immunosuppressant for attenuating inflammation-induced disorders [43].

*Bradysia odoriphaga* is one of the main insect pests influencing Northern China’s Chinese chive that results in 30–80% loss in production and attacks 20–30% of Chinese chives [74]. It also feeds on other plant species such as garlic, Welsh onion, cucumber, Chinese cabbage, and lettuce, as well as mushroom sheds causing production losses. Its larvae gather in the stems and roots of the plant making its control hard with common strategies. Generally, organophosphates are the principal tool for controlling Chinese chives pests [75].

Wu et al. separated and characterized two new sesquiterpenes, 9β-hydroxy-1βH,11αH-guaia-4,10(14)-dien-12,8α-olide (**15**) and 9β-hydroxy-1βH, 11βH-guaia-4,10(14)-dien-12,8α-olide (**16**), along with four known related analogs, **33**, **47**, **50**, and **55**, from the *C. abrotanoides* fruits’ MeOH extract. These metabolites had antifeedant effectiveness versus *Plutella xylostella* third-instar larvae (EC_50_ ranged from 19.84 to 97.94 mg/L). Furthermore, they displayed stomach–contact combination toxicity versus *B. odoriphaga* fourth-instar larvae (LD_50_s 18.71–319.67 mg/L), whereas **15** had the potent activity, revealing its potential use as a natural insecticide. The results indicated that the β-C-11 methyl group in **15** and **47** made them more active than **16** and **50** that have α-C-11 methyl group [44].

**Figure 5:**
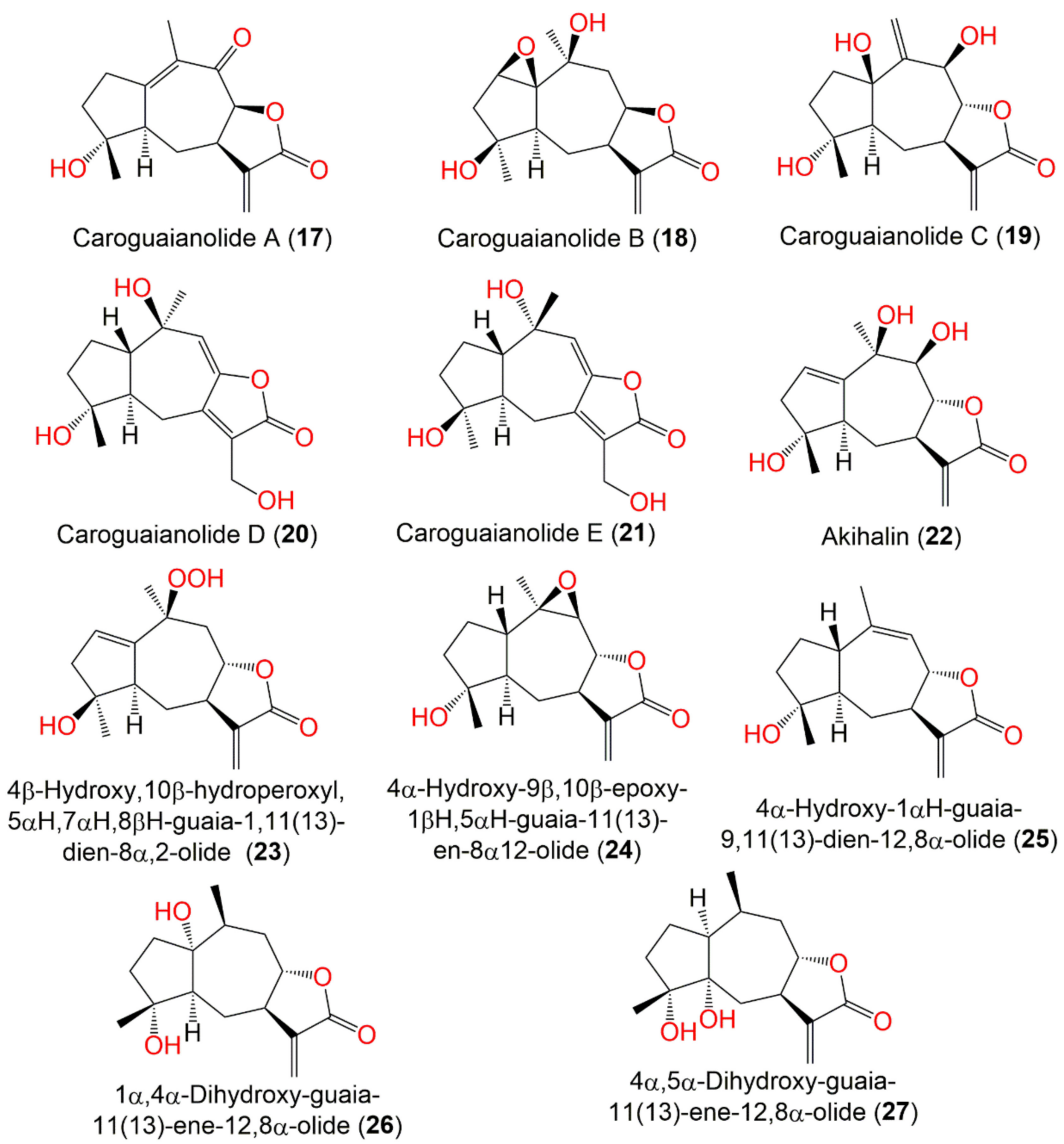
Guaianolide sesquiterpenes (**17**–**27**) reported from *C. abrotanoides*.

Wang et al. purified five new guaiane-type sesquiterpene lactones, caroguaianolides A–E (**17**–**21**), along with formerly reported **10**, **12**, **22**–**25**, **30** and **32** from the EtOAc fraction of 95% EtOH extract (Figure 5).

**Scheme 2:**
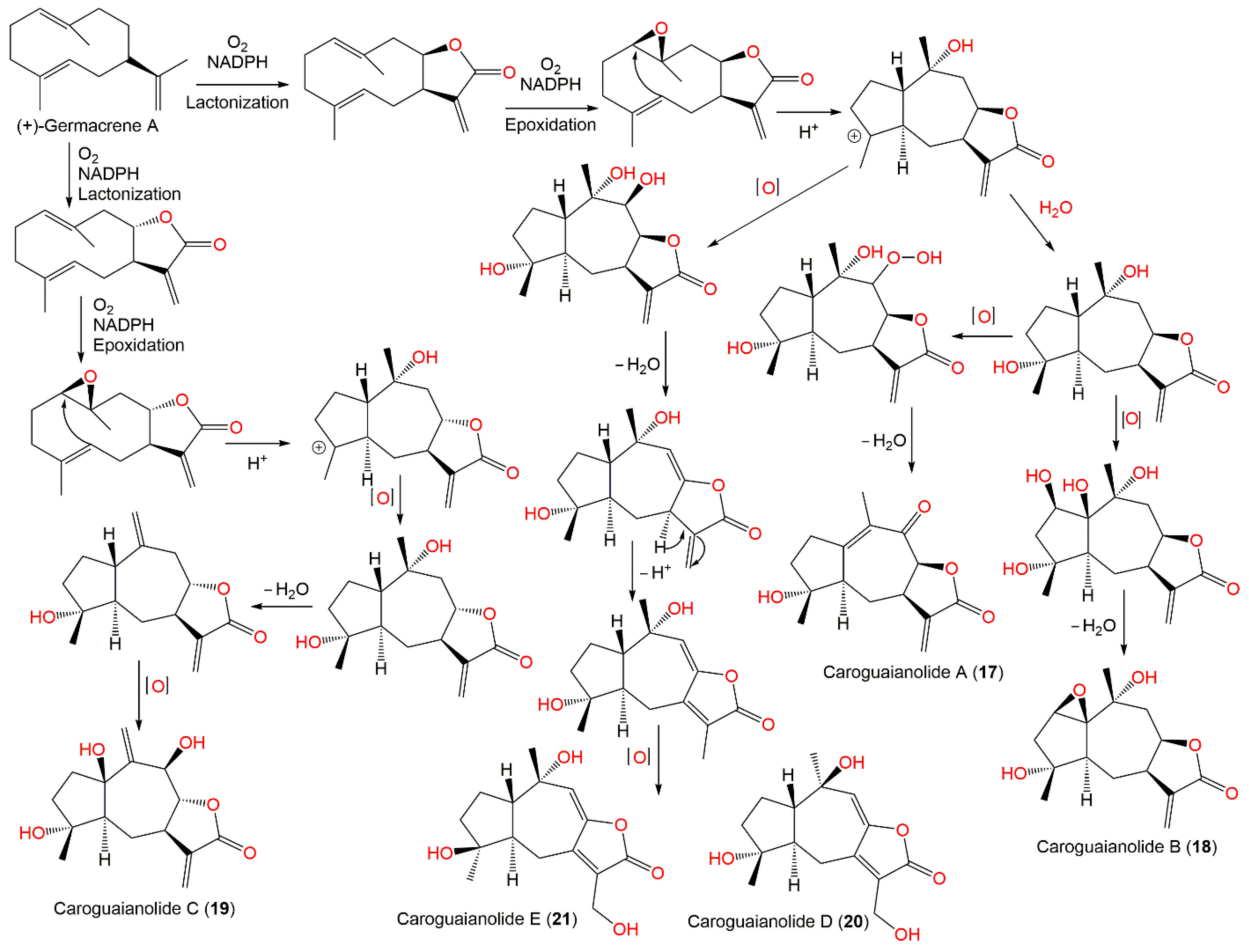
Biosynthetic pathway of caroguaianolides A–E (**17**–**21**) [51].

In the MTT assay, **17**–**19**, **22**, **23**, **31**, and **32** demonstrated marked cytotoxic potential (IC_50_ ranged from 2.67 to 12.34 μM) versus HGC-27 and MDA-MB-231 cell lines, compared to mitomycin C (IC_50_ 6.68 and 4.56, respectively). It was found that **32**, **17**–**19**, **22**, **23**, and **25** with α-methylene γ-lactone moiety had strong effectiveness; however, **20** and **21** without this moiety displayed weak activity, indicating that α-methylene γ-lactone moiety was substantial for guaiane sesquiterpenes’ cytotoxic effectiveness [51]. These metabolites were postulated to be derived from (+)-germacrene A as illustrated in Scheme 2.

**Figure 6:**
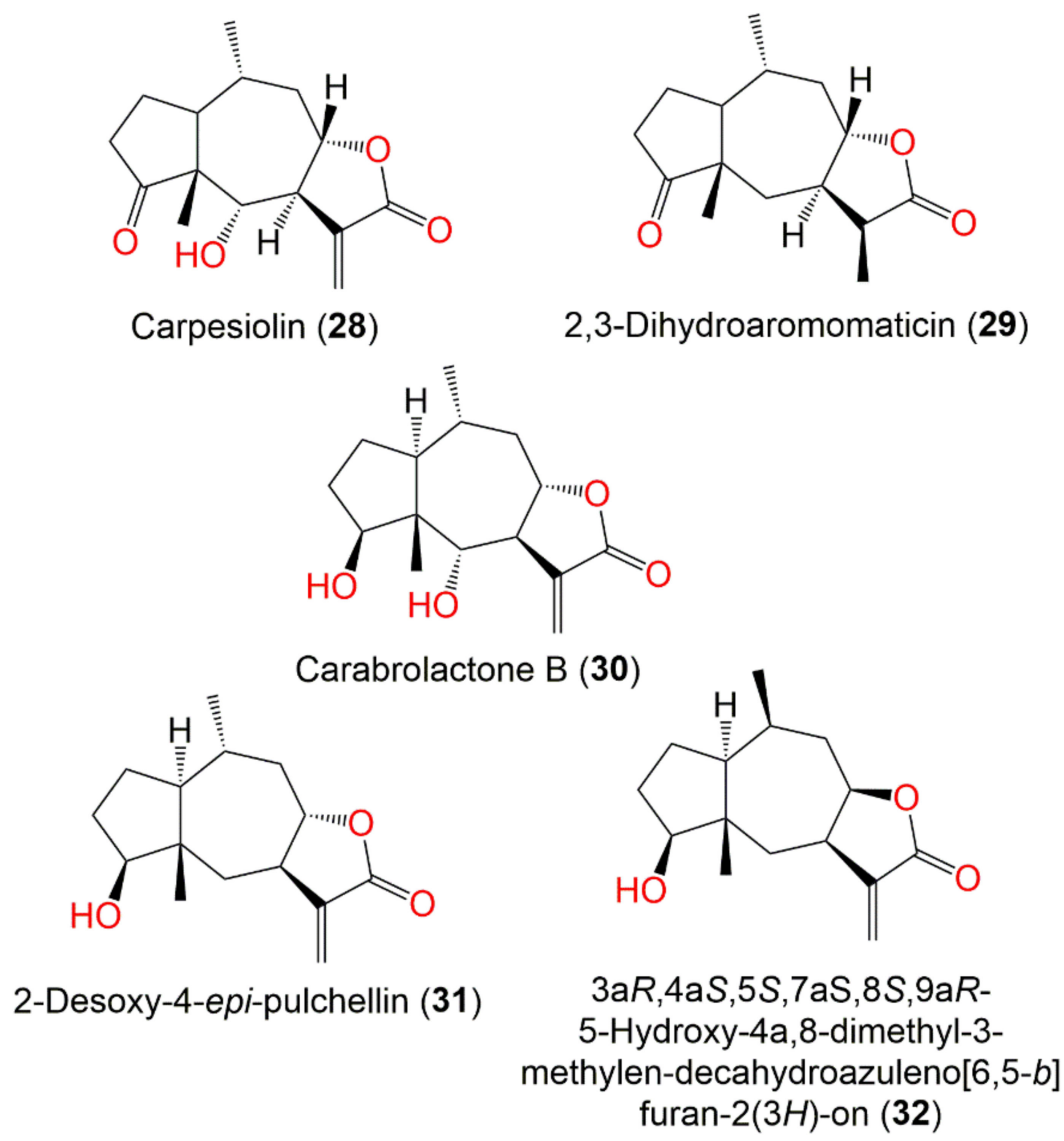
Pseudo-guaianolide sesquiterpenes (**28**–**32**) reported from *C. abrotanoides*.

In 2018, Hu et al. characterized new sesquiterpene lactones, **46**, **58**, and **59**, along with **13**, **31**, **37**, **55**, and **56** from the whole plant EtOAc fraction (Figure 6). Compound **46** was similar to **37** except for the existence of a C-12 methyl group instead of the exocyclic methylene in **37**. On the other side, **58** and **59** were analogs of **56** with a C-4 palmitoyloxy and linoleoyl group, respectively, instead of C-4 OH in **56**. Only **58** possessed cytotoxic influence versus the HL60 cells (IC_50_ 45.85 μM) in the MTS method [53].

**Figure 7:**
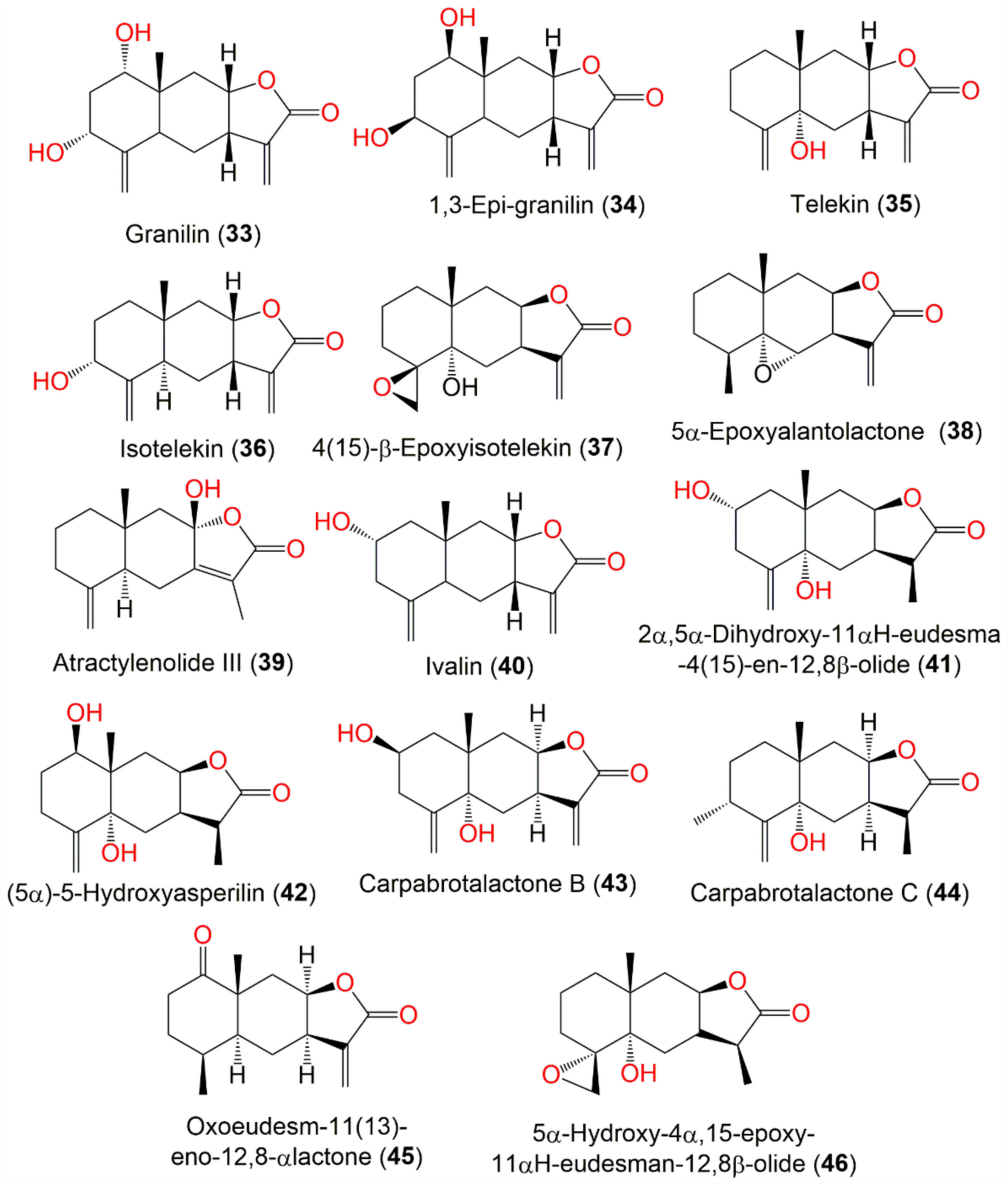
Eudesmanolide sesquiterpenes (**33**–**46**) reported from *C. abrotanoides*.

Telekin (**35**), 2α,5α-dihydroxy-11αH-eudesma-4(15)-en-12,8β-olide (**41**), and oxoeudesm-11(13)-eno-12,8α-lactone (**45**) (Figure 7) demonstrated cytotoxic potential versus HepG-2, where **35** and **45** (IC_50_s 2.95 and 4.15 μM, respectively) with γ-lactone-α-methylene moiety had more potent effects than **41** (IC_50_ 9.83 μM). These metabolites noticeably reduced the STAT3 and JAK2 mRNA expression as well as prohibited the p-STAT3 and p-JAK2 expression in the HepG-2 cells. Hence, their antiproliferative potential versus HepG-2 was due to STAT3/JAK2 signaling pathway suppression [16].

In 2019, Wang et al. reported the separation of new eremophilane sesquiterpenoids, carperemophilanes A (**53**) and B (**54**), along with **56** from the EtOAc fraction of the whole plant 95% EtOH extract [60]

**Scheme 3:**
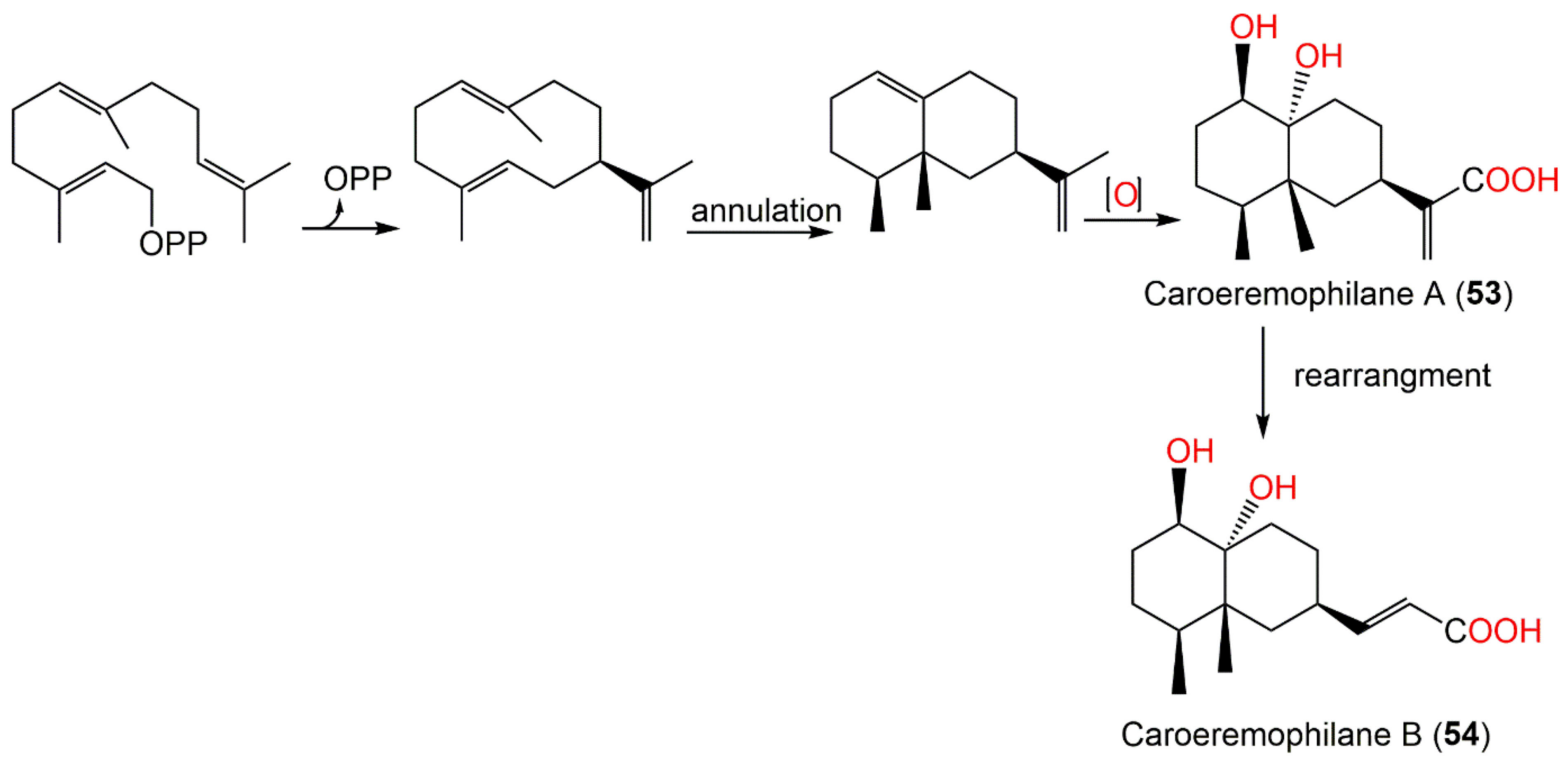
Biosynthetic pathway of carperemophilanes A and B (**53** and **54**) [60].

Compounds **53** and **54** had 1R/4S/5R/7S/10R configuration. These metabolites displayed cytotoxic capacities (IC_50_ ranging from 7.45 to 37.35 μM) versus MDA-MB-231 and HGC-27 in the MTT method, whereas **56** displayed potent effectiveness (IC_50_s 7.45 and 10.27 μM, respectively). Biosynthetically, **53** and **54** are assumed to be produced from farnesylpyrophosphate (FPP) through the mevalonate pathway. Caroeremophilane A (**53**) is generated from FPP by annulation and a series of oxidation; however, a rare rearrangement and turning of the isopropyl group into n-propyl result in the formation of caroeremophilane B (**54**) (Scheme 3) [60].

**Scheme 4:**
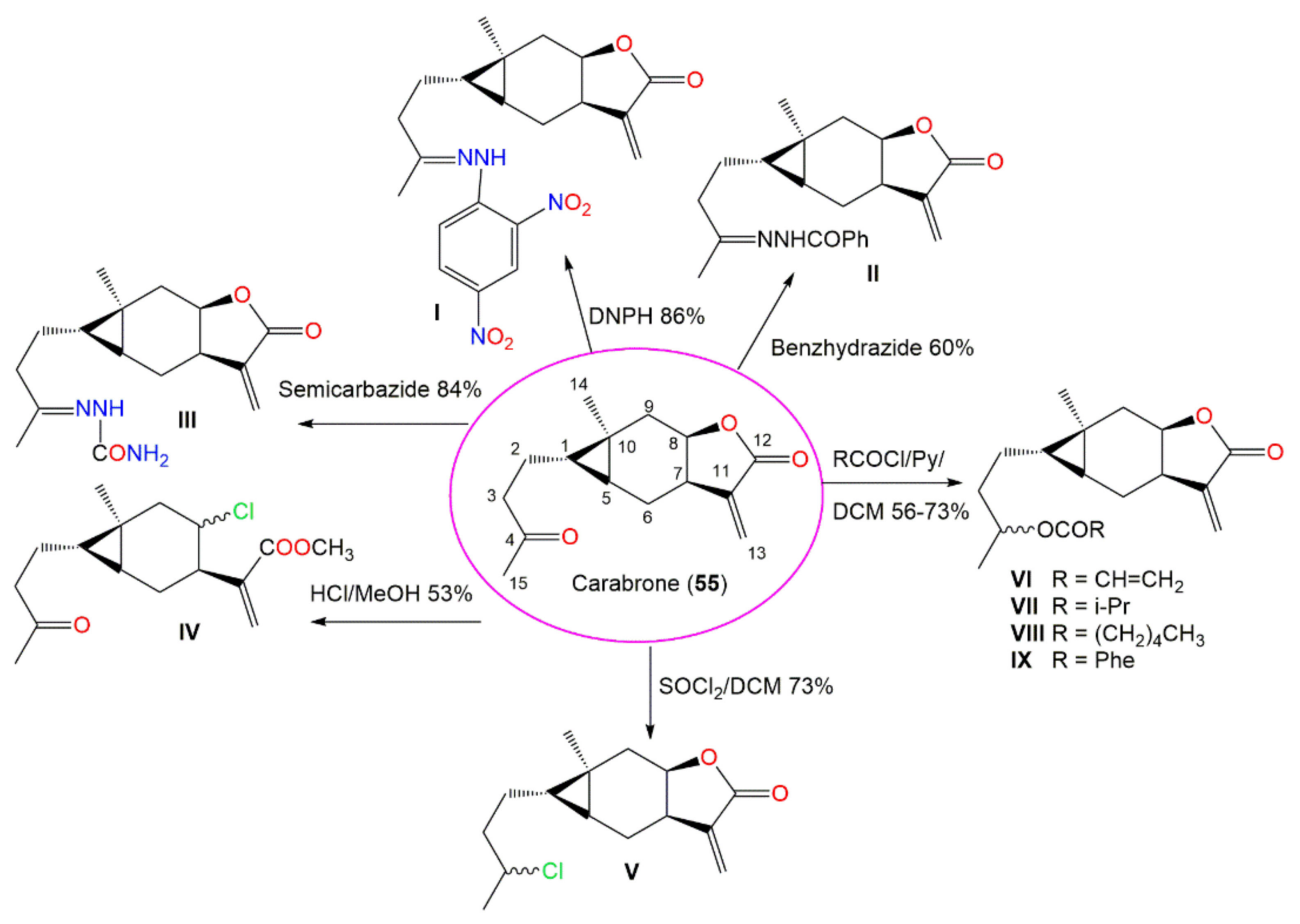
Synthetic pathway of carabrone derivatives **I**–**IX** from carabrone (**55**) [69].

Carabrone (**55**), a sesquiterpene lactone with cyclopropane moiety possessed noticeable in vitro antifungal potential (EC_50_ 7.1 μg/mL) versus *Colletotrichum lagenarium* Ell. et. Halst compared with chlorothalonil (EC_50_ 0.75 μg/mL) in the spore germination method. It was found that C-4 carbonyl group and Δ^11,13^ double bond are the active sites as evident by the synthesis of new (**I**–**IX**) derivatives of **55** (Scheme 4). It was demonstrated that the γ-lactone was substantial for the effectiveness and introduction of hydrazone substituents at C-4, resulting in more potent derivatives [69].

**Figure 8:**
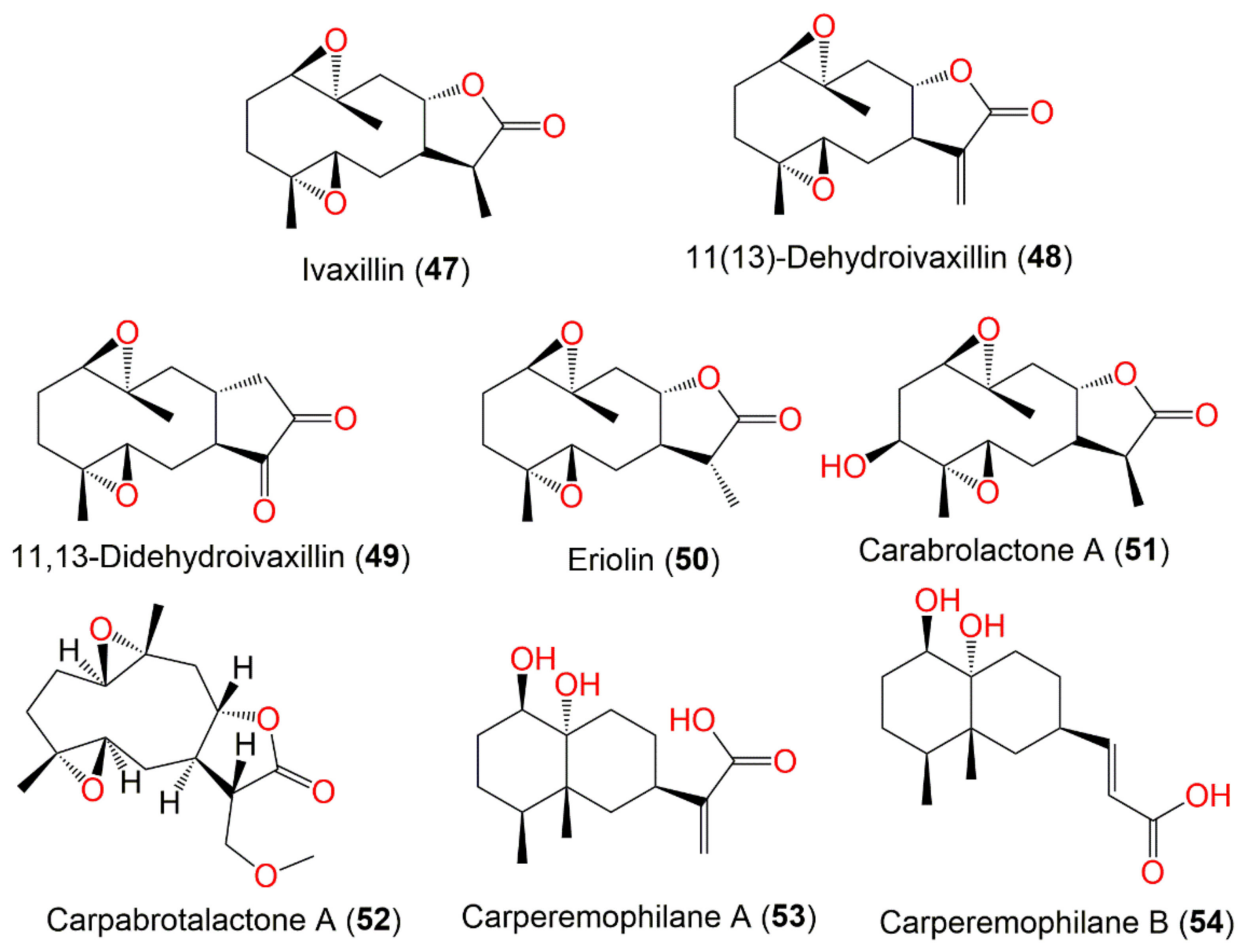
Germacranolide (**47**–**52**) and eremophilanoide (**53** and **54**) sesquiterpenes reported from *C. abrotanoides*.

Two new sesquiterpenes, named carpabrotalactones A (**52**) and B (**43**), together with **26**, **27**, **41**, **42**, **48**, and **50** were purified from the EtOAc fraction of the herb’s 70% EtOH extract [54]. Compounds **43** and **52** had 2R/5R/7S/8R/10R and 1R/4R/5R/7R/8S/10R/11S configuration based on X-ray analysis (Figure 8). These sesquiterpenes possessed cytotoxic potential (IC_50_ ranged from 7.8 to 47.4 μM) versus ISK, A549, Sw620, HeLa, Caco-2, and RBE cell lines in the CCK-8 assay. Moreover, **26**, **27**, **43**, **48**, and **50** displayed notable anti-H1N1 effectiveness (IC_50_ ranged from 0.4 to 16.4 μM), whereas **26** and **27** revealed the potent effect compared to oseltamivir (IC_50_ 0.05 μM), suggesting their potential as anti-H1N1 agents [54].

Carpabrotalactone C (**44**) revealed two alicyclic ring-containing sesquiterpene lactones and had 3R/5S/7R/8R/10R/11S configuration. It exhibited cytotoxic potential versus MCF-7, A549, SW480, and SMMC-7721 cell lines (IC_50_s 11.46–23.68 μM) in the CCK-8 assay compared to cisplatin (IC_50_s 5.1–12.77 μM). Meantime, it had weak inhibition potential (IC_50_ 63.23 μM) on NO release induced by LPS in RAW 264.7 cells relative to dexamethasone (IC_50_ 3.61 μM) [58].

**Figure 9:**
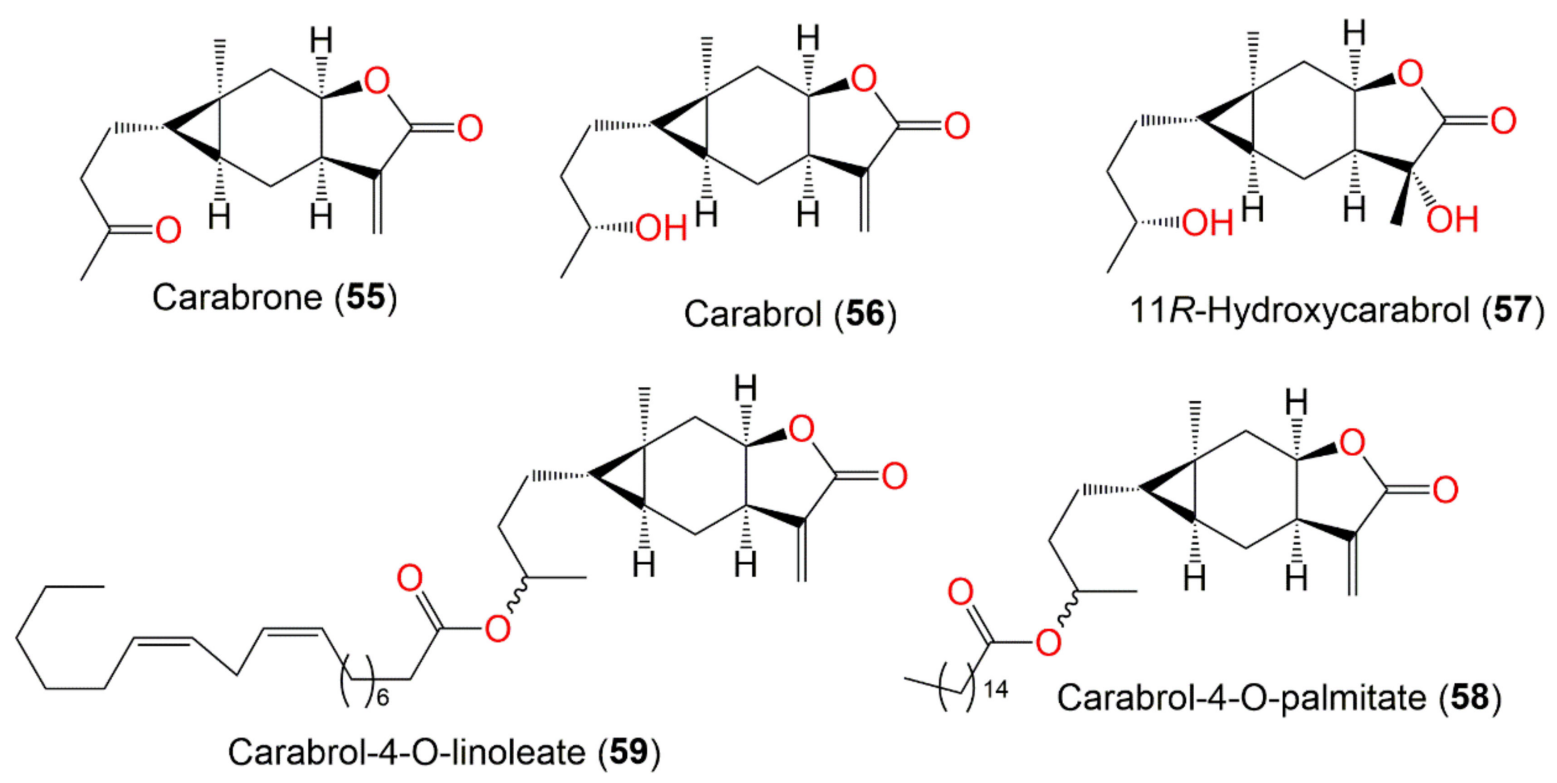
Sesquiterpenes with cyclopropane ring (**55**–**58**) reported from *C. abrotanoides*.

Yang et al. separated **10**, **29**–**31**, **34**–**40**, **42**, **48**, and **56** from the PE and EtOAc fractions of the whole plant’s 95% MeOH extract (Figure 9) that were assessed for cytotoxic potential towards A549, MDA-MB-231, HepG2, CNE2, HCT116, and NCM460 in the MTT assay. Compound **38** (IC_50_ 2.73–7.21 μM) demonstrated the powerful effectiveness versus all cell lines. It was found to effectively induce G2/M cell cycle arrest and ROS accumulation, leading to cancer cell apoptosis. Interestingly, **35** and **38** activated protective autophagy and induced lysosomal biogenesis (173.2% and 163.7%, respectively) [52].

Additionally, sesquiterpenes dimers are reported from *C. abrotanoides*. Biosynthetically, they possess uncommon carbon skeletons that are derived via the coupling of two monomeric sesquiterpene moieties. These compounds have attracted remarkable research interest for their diversified and peculiar structural features with complicated linking patterns and multiple chiral centers.

**Figure 10:**
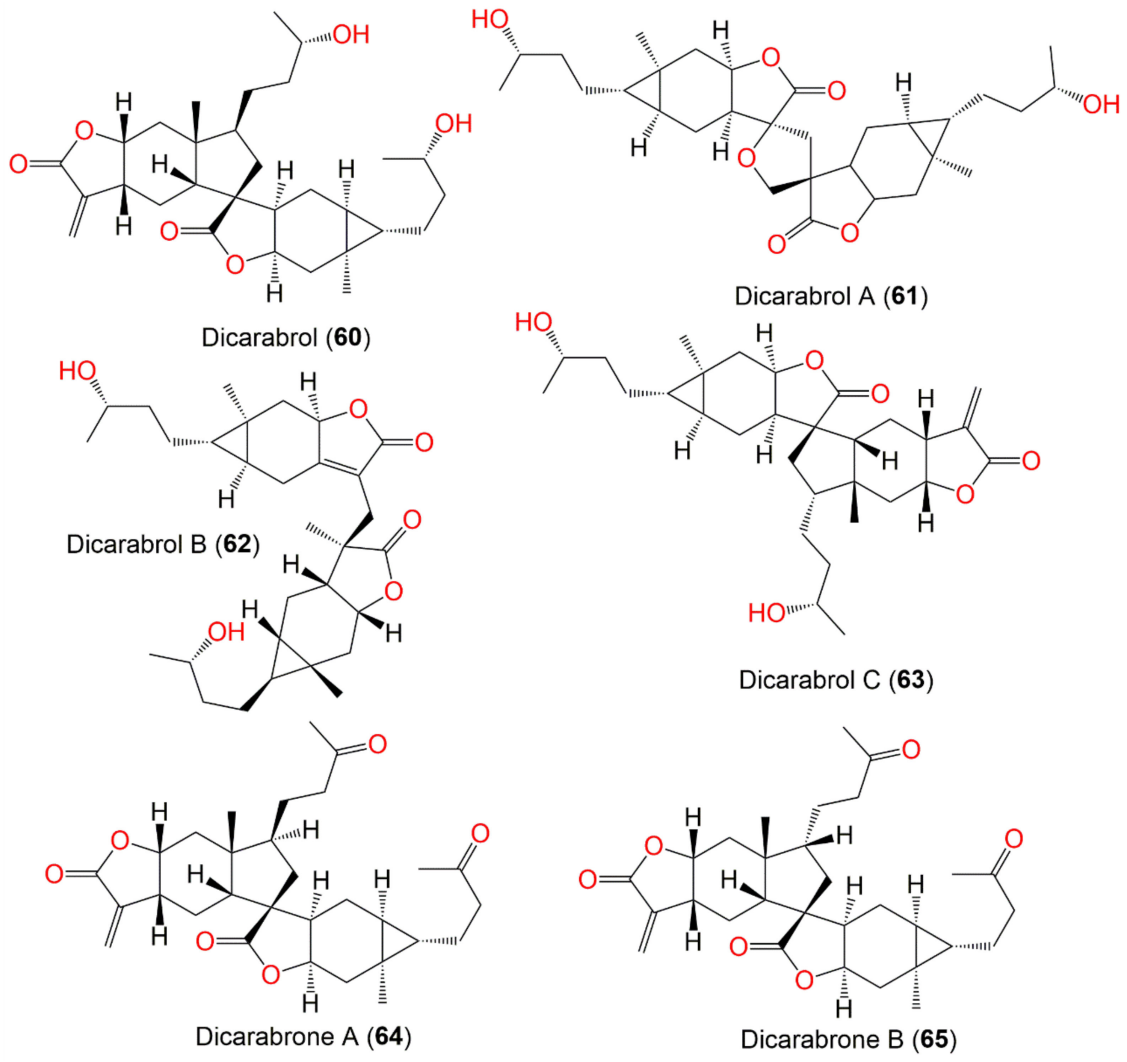
Dimeric sesquiterpenes (**60**–**65**) reported from *C. abrotanoides*.

A new dimeric sesquiterpene, dicarabrol (**60**), in addition to **31**, **48**, and **56** were purified from *C. abrotanoides* whole plant EtOAc fraction (Figure 10). Compound **60** was 2R,3R,4R,6R,7S,10S,11R,12R,14R,15R,20S,23S,28S-configured and featured two sesquiterpene lactone units connecting through a cyclopentane ring. These metabolites revealed potent in vitro cytotoxic capacity versus the K562, MCF-7, Hela, DU145, U937, H1975, SGC-7901, A549, MOLT4, and HL60 cell lines (IC_50_ ranged from 0.10 to 46.7 μM), compared with taxol (IC_50_s 2.1–9.8 μM), whereas **60** had powerful effectiveness than taxol versus all cell lines. Moreover, compounds **31**, **48**, and **60** had antiviral potential versus H1N1 (IC_50_s 10.8–45.5 μM) and H3N2 (IC_50_s 11.6–47.3 μM) compared to osehamivir (IC_50_s 0.025 and 0.015 μM, respectively) in the CPE inhibition assay and marked antimycobacterial capacity (MICs 3.7, 6.0, and 7.6 μM, respectively) versus *M. tuberculosis*, relative to INH (isoniazid, MIC 2.0 μM) in the GFPMA (green fluorescent protein microplate assay. All compounds possessed no COX-1 and COX-2 inhibition [56].

Jie-Wei et al. reported the separation of two new dimeric sesquiterpenes of carabrane class, dicarabrols B (**62**) and C (**63**), and a new carabrane sesquiterpene, 11*R*-hydroxycarabrol (**57**) (Figure 8) [62]. Compound **62** has a C_30_ novel dimeric skeleton, possessing a methylene-bridged two sesquiterpene moieties, whereas **63** displayed unique cyclopentane linkage. Compound **63** revealed selective cytotoxic effectiveness versus HL-60 (IC_50_ 3.7 μM) and had a weak influence on A549 cells (IC_50_ > 20 μM), while **62** and **57** were inactive, suggesting that γ-lactone-α-methylene moiety had a role in activity [62].

Wu et al. characterized a pair of dimeric sesquiterpene epimers, dicarabrones A and B (**64** and **65**), from the whole plant CHCl_3_ fraction. Their skeletons are characterized by two sesquiterpene-lactone moieties, connected via a cyclopentane ring. They are epimers at C-1′ with 1S,5S,7R,8R,10R,11R,1′S,5′R,7′R,8′R,10′R and 1S,5S,7R,8R,10R,11R,1′R,5′R,7′R,8′R,10′R configurations, respectively, relying on ROESY and X-ray results. Compounds **64** and **65** (IC_50_s 9.1 and 8.2 μM, respectively) displayed selective cytotoxic potential versus HL-60 cells and weak effectiveness (IC_50_ > 10 μM) versus A549 cells in the MTT and SRB assays, respectively [64].

**Scheme 5:**
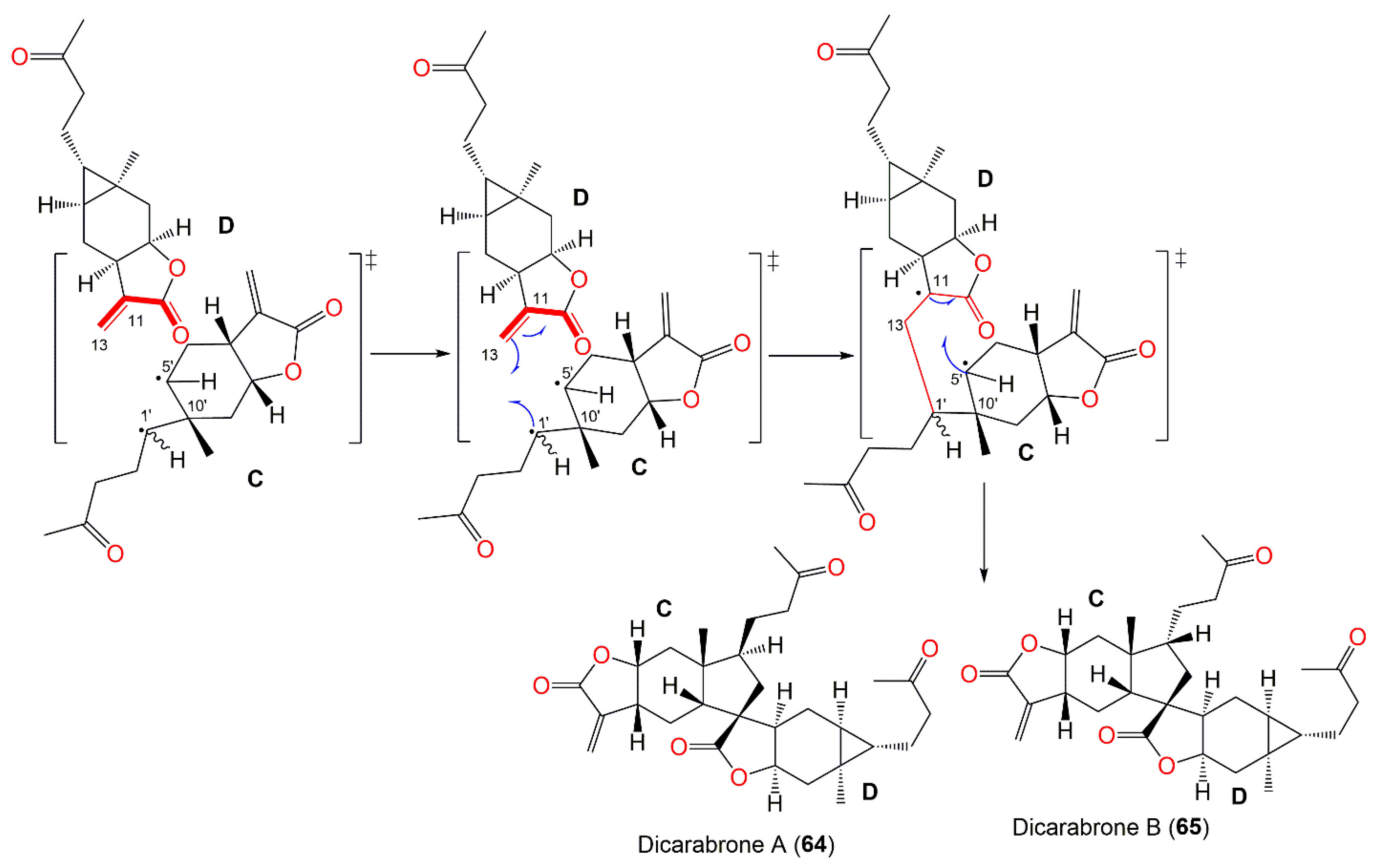
Biosynthetic pathway of dicarabrones A (**64**) and B (**65**) [64].

It was postulated that the cyclopentane unit in **64** and **65** could be generated from a [3+2] cyclo-addition reaction beginning from a three-membered ring in one molecule and a double bond in the other (Scheme 5) through radical intermediates. The cleavage of the bond between C-5′ and C-1′ forms a radical-intermediate **C** having C-5′ already radical that is then trapped with the double bond in **D**. Due to steric hindrance in the structure, C-1′ firstly forms a new bond with C-13, and then C-5′ forms another bond with C-11. Hence, a five-membered ring fuses the two monomers **C** and **D**. Additionally, the free rotation of the side chain around C-1′ resulted in two possible configurations at C-1′ [64].

**Scheme 6:**
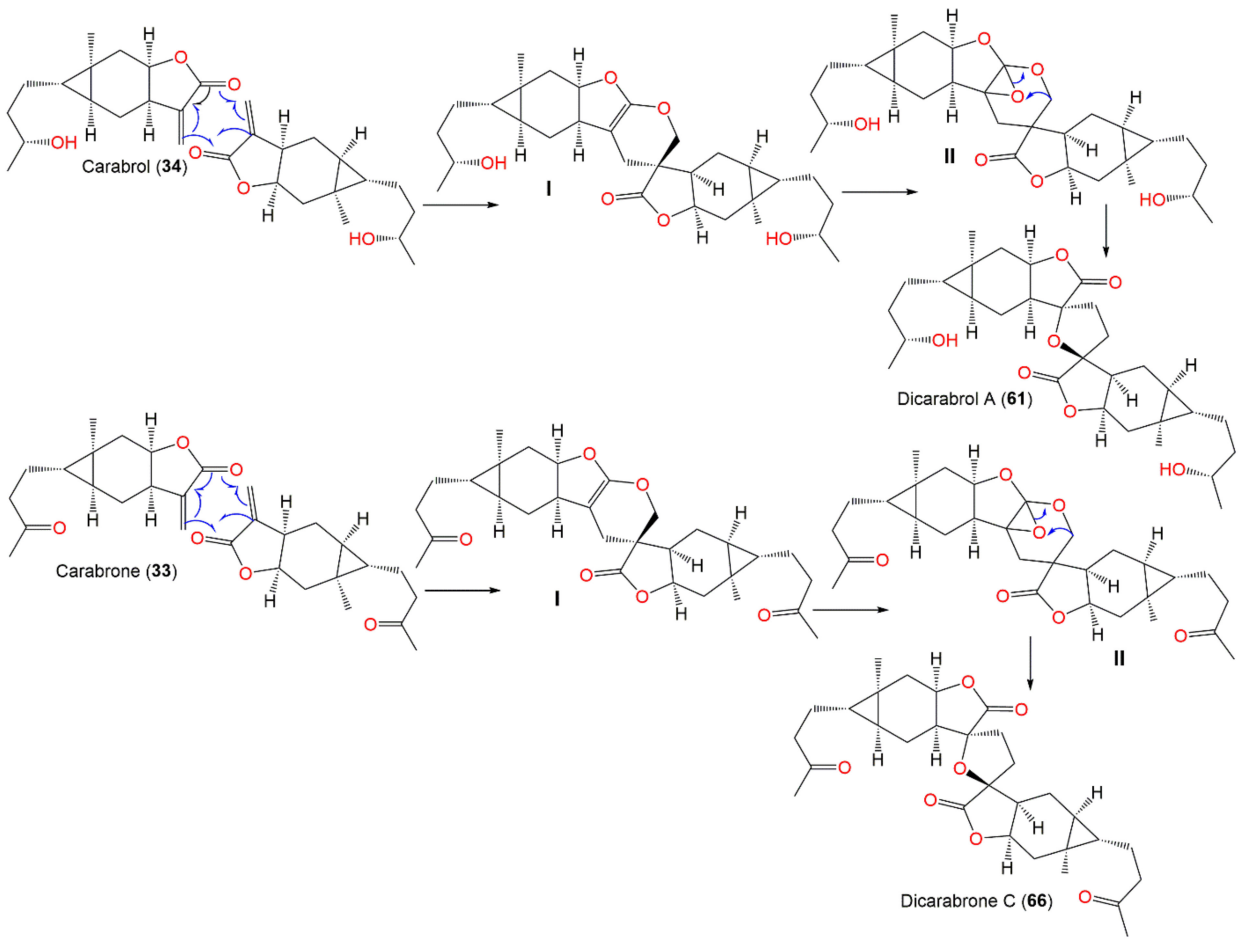
Biosynthetic pathways of dicarabrol A (**61**) and dicarabrone C (**66**) [63].

The investigation of the whole plant’s 95% EtOH extract afforded rare dimeric sesquiterpenes, dicarabrol A (**61**), dicarabrone C (**66**), and dipulchellin A (**74**). Compounds **61** and **66** featured an unwonted skeleton with two linked carabranolide moieties via a spiro-tetrahydrofuran ring that was assumed to be generated by a [4+2] cycloaddition; however, **74** is a product of [3+2] cycloaddition of a carabranolide moiety and guaianolide moiety connecting via a cyclopentane ring. Their configurations were established as 1S/5S/7S/8R/10R/11S/1′S/5′S/7′R/8′R/10′R/11′R for **61** and **66** and 1S/4S/5S/7R/8S/10R/11S/1′R/4′S/5′R/7′R/8′R/10′R for **74**. These metabolites revealed selective cytotoxic potential versus HL-60 cells (IC_50_s 8.7, 8.2, and 8.9 μM, respectively), while they had no activity versus A549 cells (IC_50_ > 10 μM) in the MTT assay [63]. Biosynthetically, it was proposed that [4+2] Diels–Alder cycloaddition is the main step for **61** and 66 biosyntheses (Scheme 6). Carabrone (**55**) and carabrol (**56**) could be the parent compounds for their biosynthesis. The double bond (C11′=C13′) of one carabrone/carabrol undergoes [4+2] Diels–Alder cycloaddition with the α,β-unsaturated carbonyl (C13=C11–C12=O) of another carabrone (**55**)/carabrol (**56**) to yield an intermediate I with a dihydropyran ring. Then, the enzymatic oxygenation of the dihydropyran double bond produces an oxirane ring in II. Lastly, two C–O bonds displace each other to form the tetrahydrofuran ring in dicarabrone C (**66**)/dicarabrol A (**61**) [63].

**Figure 11:**
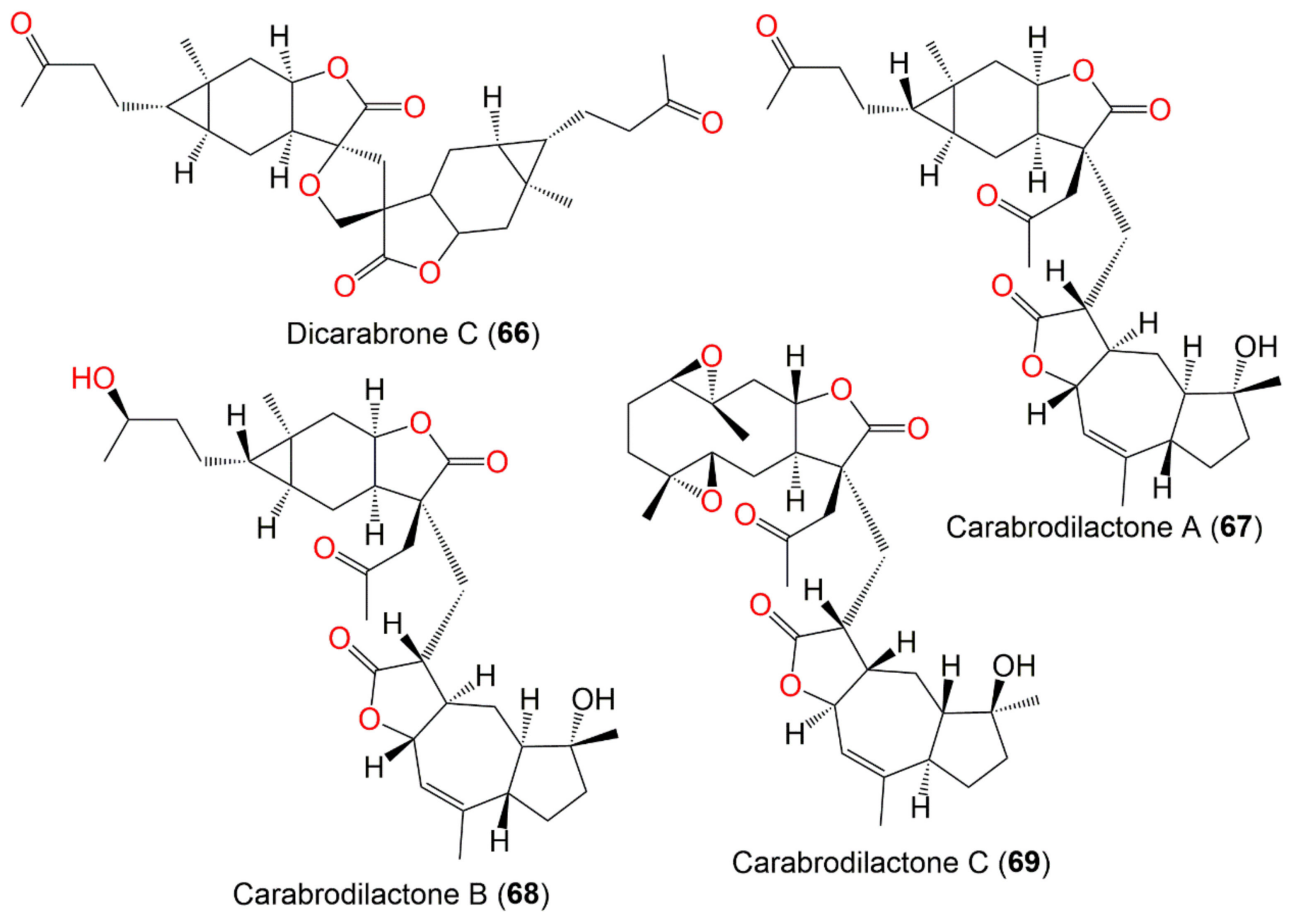
Dimeric sesquiterpenes (**66**–**69**) reported from *C. abrotanoides*.

**Figure 12:**
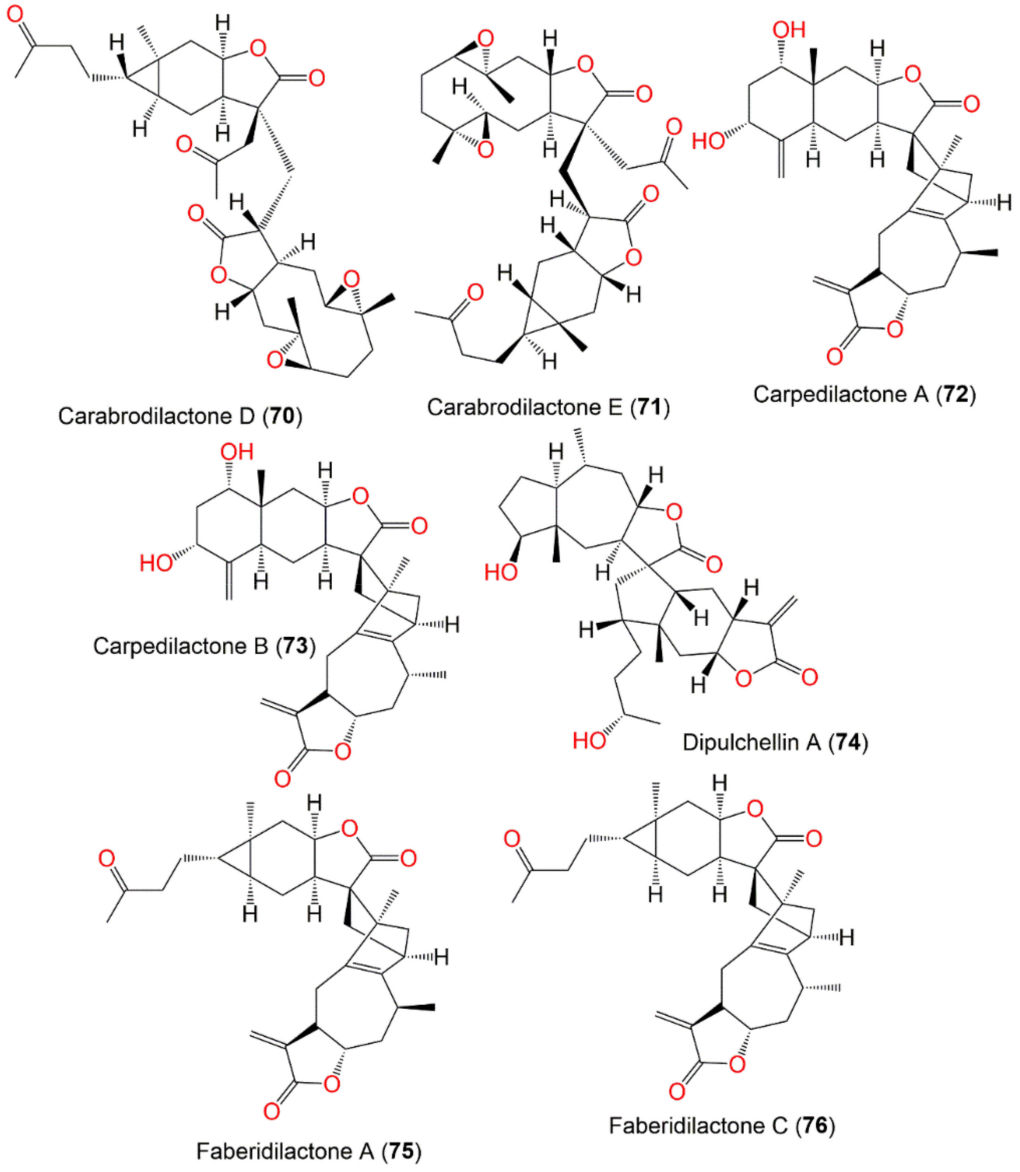
Structures of dimeric sesquiterpenes (**70**–**76**) reported from *C. abrotanoides*.

In 2020, Yang et al. purified five uncommon C15/C17 dimeric sesquiterpenes, carabrodilactones A–E (**67**–**71**), along with formerly reported C15/C15 dimeric analogs, **72**, **73**, **75**, and **76**, from the EtOAc fraction of 70% EtOH of the whole plant (Figure 11 and Figure 12). Compounds **67**–**71** displayed tailed acetyl linked to the C-13 position and flexible C-13′/C-11-linked single bond among the two sesquiterpene moieties [65]. They were evaluated for their cytotoxic potential versus MDA-MB, A549, BEL7404, and HCT116 cells using the MTT assay. Among them, **67** had potent effectiveness versus these cell lines (IC_50_ ranged from 3.08–8.05 μM); however, the others possessed weak potential (IC_50_ 27.70–101.34 μM). It was demonstrated that the α-ethylene-γ-lactone group’s exo-ethylene, C-4 carbonyl of carabrone unit, and two sesquiterpene units’ spatial orientation were crucial for the activity. Moreover, **75** and **76** were found to have cytotoxic potential versus K562, CCRF-CEM, HCT116, and HL-60 cells (IC_50_ ranged from 2.63 to 8.50 µM) in the MTT assay [70].

**Scheme 7:**
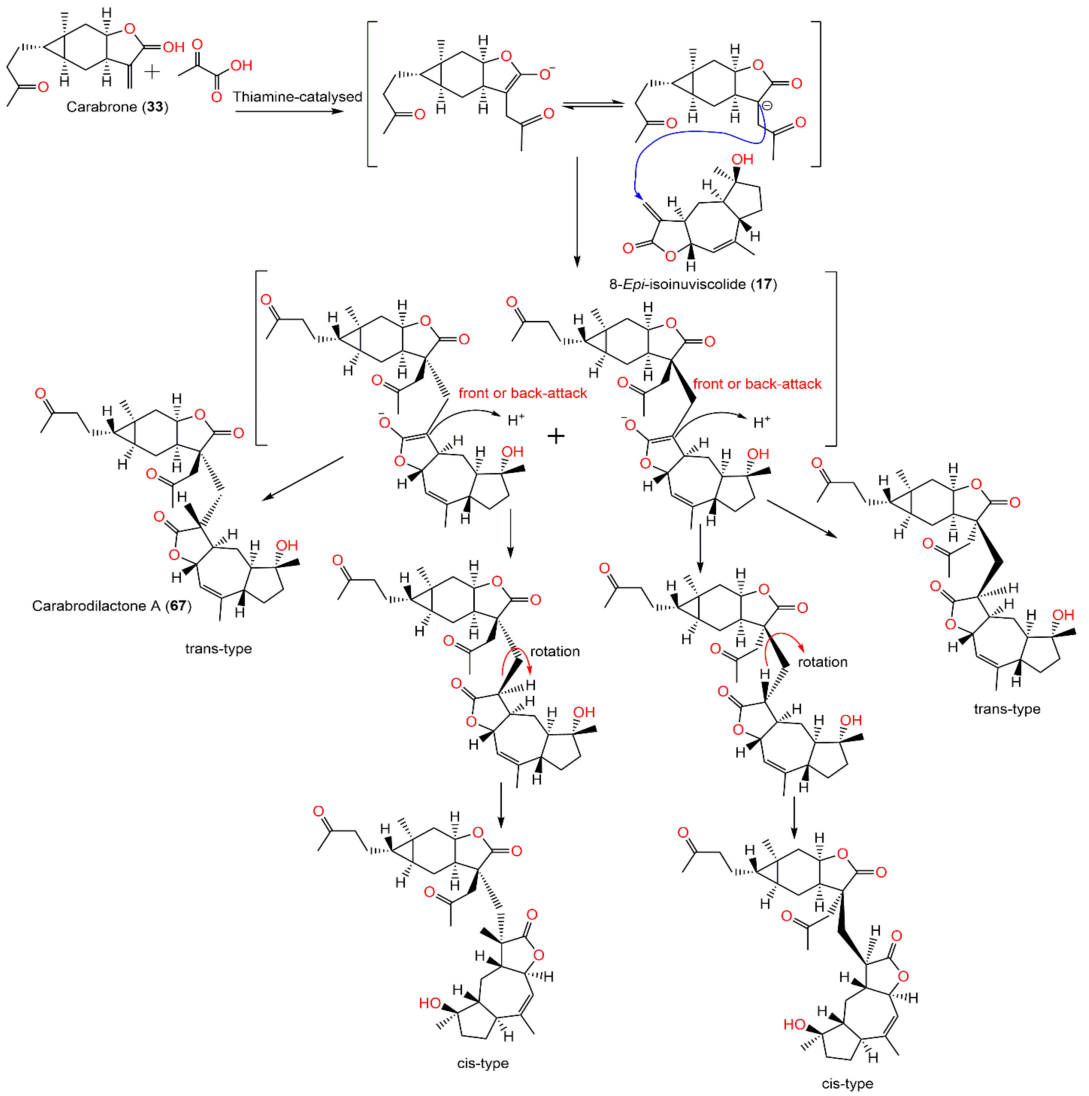
Biosynthetic pathway of carabrodilactones A–E (**67**–**71**) [65].

Biosynthetically, these C15/C17 metabolites were assumed to be generated through Stetter and Michael’s addition reactions. Firstly, the acetaldehyde anion is formed by umpolung of pyruvic acid through catalysis by thiamine. After that, it attacks the first C15 sesquiterpene lactone exoethylene to generate the C17-enolate anion at C-11 by Stetter reaction. Thenceforward, the Michael addition reaction of enolate with the α,β-unsaturated bond of the other lactone moiety yields four possible C17/C15 stereo-isomeric dimers with a C-13′/C-11 C-C bond among the two sesquiterpene moieties [65] (Scheme 7).

**Scheme 8:**
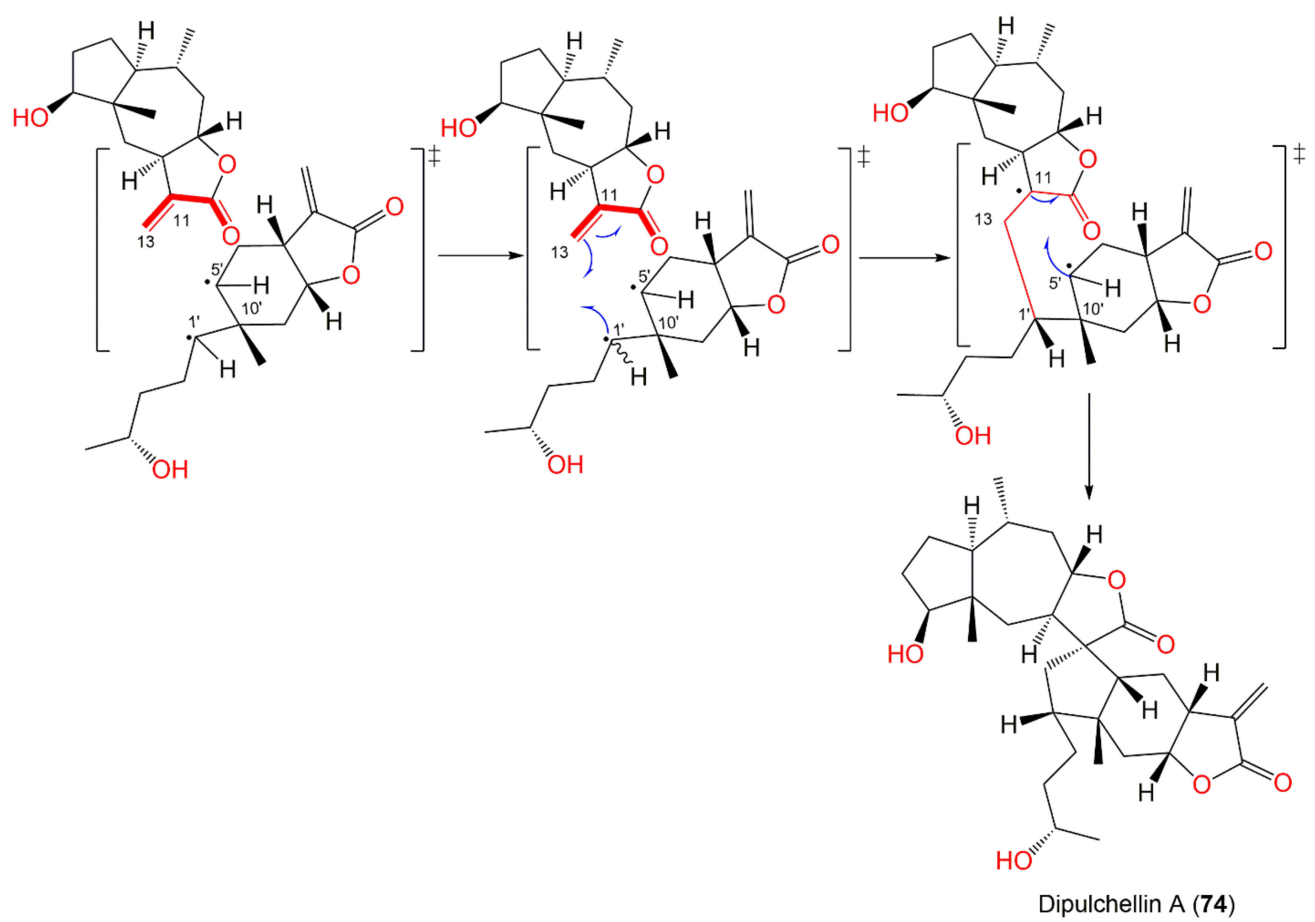
Biosynthetic pathways of dipulchellin A (**74**) [63].

It was proposed that Dipulchellin A (**74**) be biosynthesized from [3+2] cycloaddition as reported in **64** and **65** C-5′-C4′ bond. The cyclopentane ring possibly originated from a [3+2] cycloaddition of a cyclopropane ring of carabrone (**55**) monomer and the double bond of 2-desoxy-4-epi-pulchellin (**31**) (Scheme 8) via radical intermediates. The C-5′- C-1′ bond is cleaved to form a radical intermediate, and the C-5′ radical is then trapped with the double bond. Due to steric hindrance in the structure, C-1′ forms a new bond with C-13 first, and subsequently C-5′ forms another bond with C-11. Thus, a cyclopentane ring is introduced to link the two monomers [63].

### 5.3. Other Metabolites and Volatile Constituents

Compounds **77**–**80** were separated from petroleum ether extract of the whole plant and evaluated for their cytotoxicity (Conc. 40 μM) versus HL60, SMMC7721, A549, SW480, and MCF7 cell lines. Only **79** had moderate effectiveness (inhibitory rates 47, 51, and 57%, respectively) versus A549, MCF-7, and SW480 [45].

Methyl 8-[2(5*H*)-furanon-5-yl]octanoate (**81**) was 5R-configured with 2(5H)-furanone connecting with the methyl octanoate moiety at C-5. It possessed the same features as ethyl-8-[2(5H)-furanon-5-yl]octanoate formerly separated from *Diospyros maritima* with less methylene [76]. It had no cytotoxic influence versus SMMC-7721, A549, SW480, and MCF-7, as well as no inhibitory potential on LPS-caused NO release in RAW 264.7 cell lines [58].

**Figure 13:**
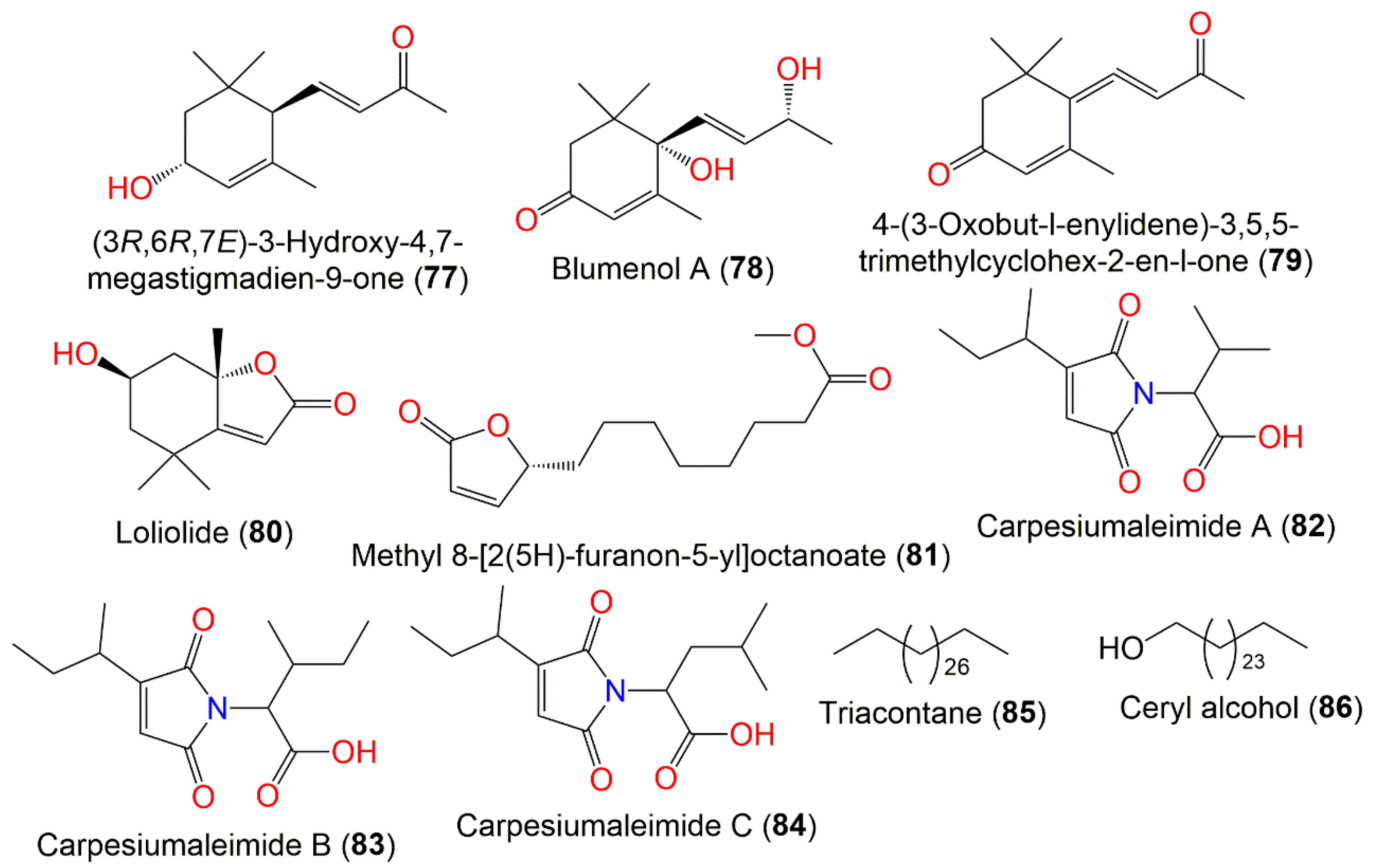
Other metabolites (**77**–**86**) reported from *C. abrotanoides*.

Wang et al. characterized carpesiumaleimides A–C (**82**–**84**), rare maleimide-bearing metabolites from the whole plant’s EtOAc fraction (Figure 13). Compound **83** was similar to **82** with isoleucine residue instead of a valine residue in **82**, while **84** exhibited a leucine residue instead of isoleucine residue in **83**. They showed weak activity (IC_50_ > 50 μM) versus MDA-MB-231 and HGC-27 in the MTT method [60].

**Figure 14:**
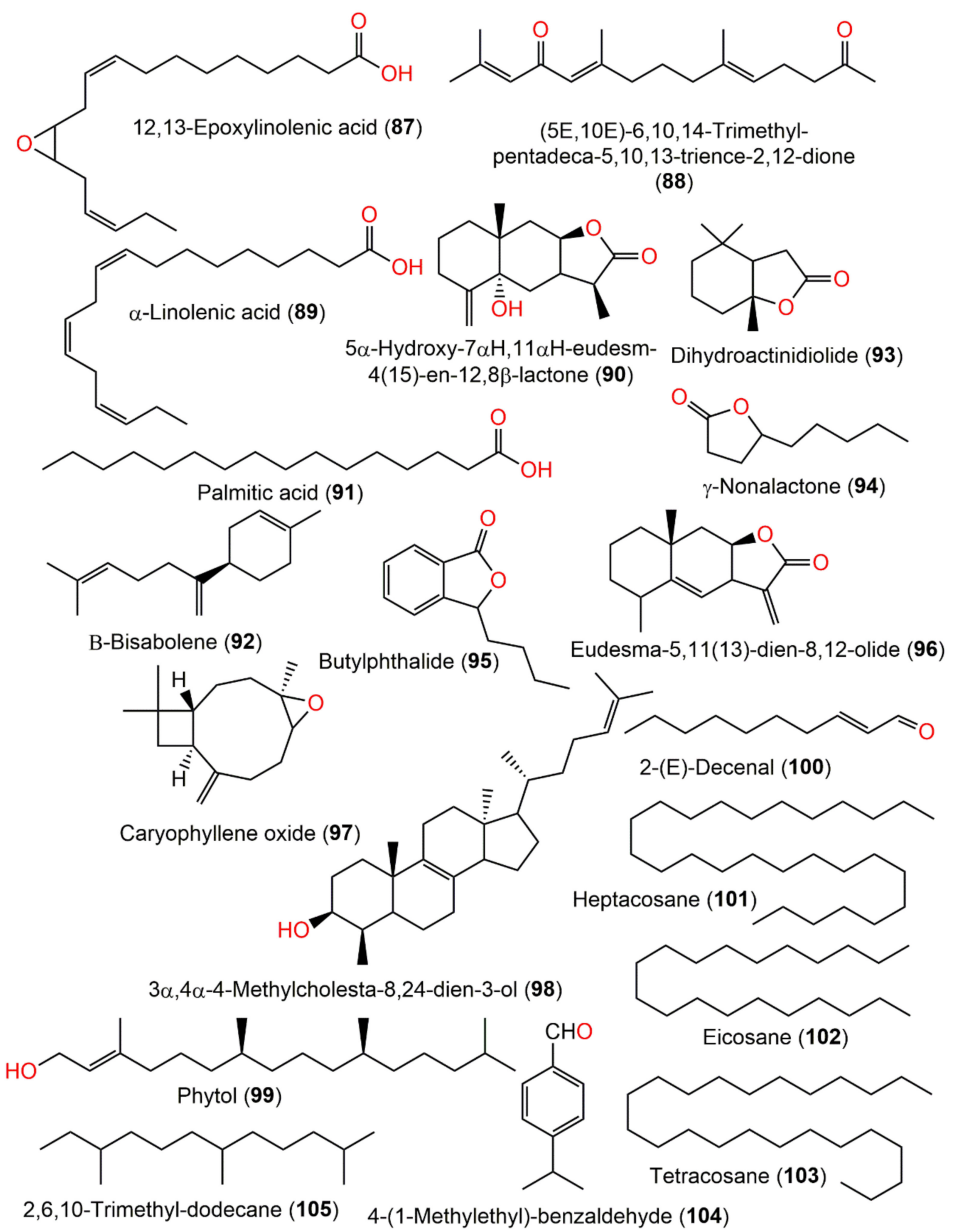
Structures of some volatile metabolites (**87**–**105**) reported from *C. abrotanoides*.

The UPLC-QTOF-MS analysis of the roots’ petroleum ether fraction led to the characterization of 12,13-epoxylinolenic acid (**87**), (5E,10E)-6,10,14-trimethyl-pentadeca-5,10,13-trience-2,12-dione, γ-linolenic acid (**88**), α-linolenic acid (**89**), 5α-hydroxy-7αH,11αH-eudesm-4(15)-en-12,8β-lactone (**90**), and palmitic acid (**91**) [17]. In 1989, Kameoka et al. analyzed the essential oil of Japanese *C. abrotanoides* herb obtained by steam distillation using GCMS, IR, GC, and ^1^H NMR. The oil had a fatty acid odor and contained β-bisabolene (**92**) and C2~C16 carboxylic and C18 fatty acids as major components. Additionally, dihydroactinidiolide (**93**), γ-nonalactone (**94**), C_3_~C_14_ aliphatic alcohols, butylphthalide (**95**), and aldehydes were identified [67] (Figure 14).

**Figure 15:**
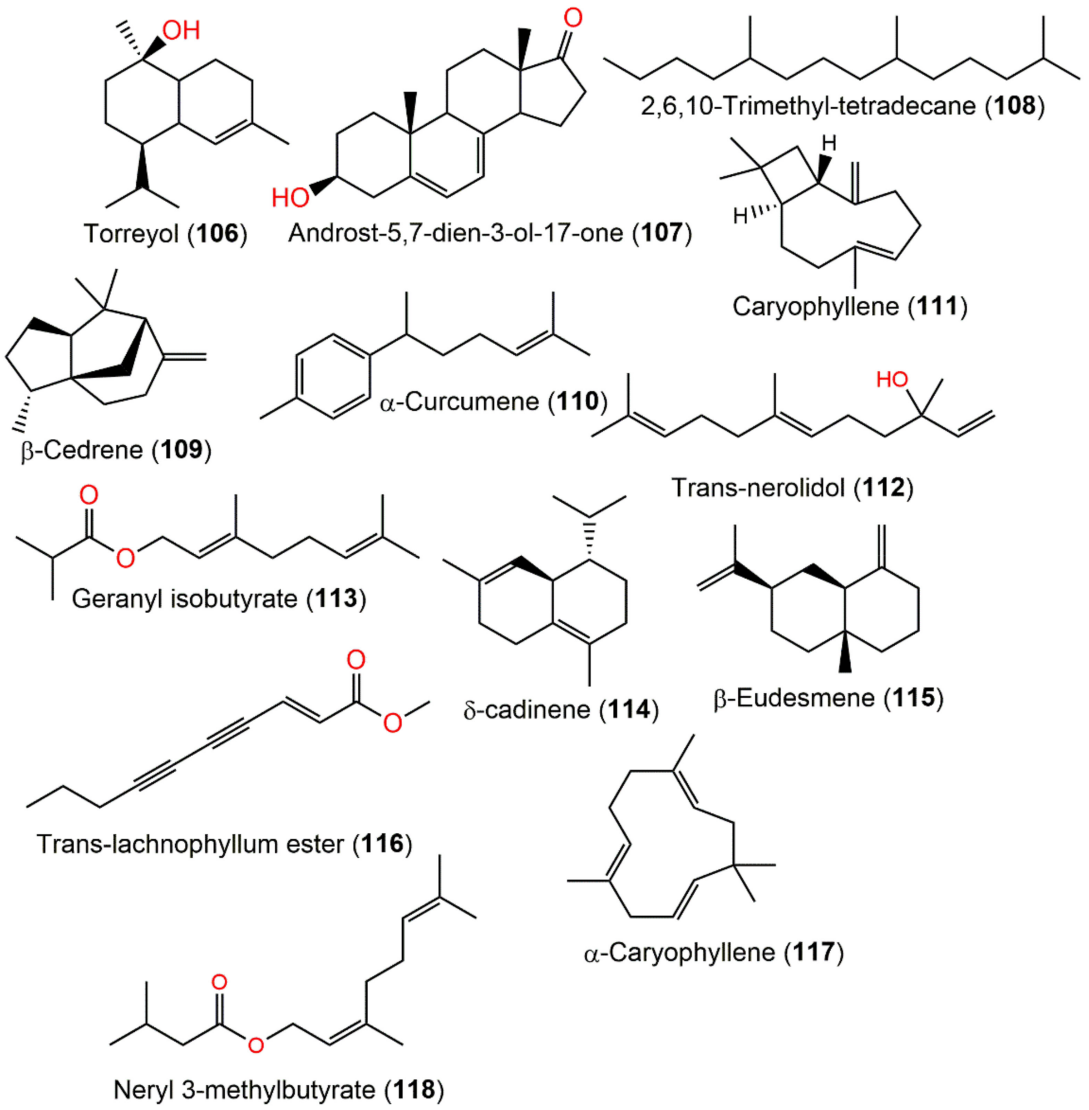
Structures of some volatile metabolites (**106**–**118**) reported from *C. abrotanoides*.

In another study, GC/MS analysis of the essential oil of the whole plant collected from Hubei province, China, that was prepared by hydro-distillation (yield 0.39% *w*/*w*) revealed the existence of sixteen compounds that represented 97.53% of the total oil, including eudesma-5,11(13)-dien-8,12-olide (**96**) (21.92%), caryophyllene oxide (**97**, 13.01%), β-bisabolene (**92**, 7.26%), 3α,4α-4-methylcholesta-8,24-dien-3-ol (**98**, 6.15%), phytol (**99**, 5.57%), 2-(*E*)-decenal (**100**, 5.56%), heptacosane (**101**, 4.84%), eicosane (**102**, 4.66%), tetracosane (**103**, 4.56%), 4-(1-methylethyl)-benzaldehyde (**104**, 4.52%), 2,6,10-trimethyl-dodecane (**105**, 4.05%), torreyol (**106**, 3.82%), androst-5,7-dien-3-ol-17-one (**107**, 3.24%), β-cedrene (**108**, 2.83%), α-curcumene (**109**), and 2,6,10-trimethyl-tetradecane (3.15%, **110**) (2.43%) [15]. On the other side, the fresh aerial parts of *C. abrotanoides* collected from the Abbottabad, Pakistan, yielded 0.04% essential oil. Its GC/MS analysis displayed 39 compounds, including caryophyllene (**111**, 24%), *trans*-nerolidol (**112**, 2%), geranyl isobutyrate (**113**, 10.63%), δ-cadinene (**114**, 8.83%), β-eudesmene (**115**, 4.67%), caryophyllene oxide (**97**, 3.89%), *trans*-lachnophyllum ester (**116**, 3.87%), α-caryophyllene (**117**, 3.5%), and neryl 3-methylbutyrate (**118**, 4.67%) (Figure 15) [32].

## 6. Toxicity Studies

Toxicity evaluation is substantial to assess the untoward effects of plants on biological systems. It is noteworthy that systemic toxicity and safety assessments are not extensively performed on this plant. Additionally, *C. abrotanoides* is formally reported to have slight human toxicity [77]. Thus, there is little evidence of this plant’s toxicity. In Chinese pharmacopeia, this plant was recorded as a mild toxic medicine [14]. *C. abrotanoides* is safe for health and its commercial preparations are commonly used for years [10]. It was reported that carpesia lactone is the main poisonous ingredient that has irritant properties and a bitter taste [78]. Its ingredients could produce certain symptoms [78] such as asdizziness, tinnitus, nausea, diarrhea, abdominal pain, colonic spasm, and weakness [79]. In Western medicine, the reported methods of its poisoning treatment include promoting vomiting, stomach washing out, grape sugar supplies, and symptomatic treatment. In Korean medicine, the herbs such as *Glycyrrhizae* Radix and Phaseoli Radiati Semen are used to counteract its poison [78]. Furthermore, Shi et al. reported a 96 h acute toxicity (LC_50_ 2.60 g/L) of *C. abrotanoides* extracts in goldfish [80]. The fruit extract also had a 24 h LC_50_ 0.76 g/L versus *Penaeus monodon* [81]. However, a study by Xia et al. proved that the fruit (Conc. 140 to 230 mg/L) had a developmental toxic influence on zebrafish larvae/embryos. It noticeably altered the embryos’ hatching rate and time and raised malformations, where the heart was the target organ. Moreover, it reduced caspase-3 potential, changed the defense enzymes (CAT, SOD, and GPX), and elevated MDA content, indicating that apoptosis and oxidative stress might be accountable for its toxic effects [82]. In clinical practice, Carpesii Fructus is mainly used as antiparasitic drugs and is usually combined with other drugs since it displayed poor efficacy as a single drug [78]. Therefore, this plant is contraindicated during pregnancy, not suitable for long time usage, and should be taken with caution for debilitated patients [79]. However, further safety studies must be carried out on *C. abrotanoides*. Future investigations are required to explore the mechanism of toxicity and to analyze and specify the extract components relevant to toxicity.

## 7. Conclusions

In this review, the traditional uses, phytoconstituents, and pharmacological activities of *C. abrotanoides* have been highlighted. The current work revealed that 118 metabolites have been purified from different parts of the plant, among them monoterpenes and sesquiterpenes are the main constituents. It is obvious that more research studies have been carried out on the aerial parts; therefore, the other plant parts should be further investigated.

It was found that the synthesis and structure modifications of certain metabolites produced more bioactive derivatives as in **13** and **33**. Hence, these constituents could be prominent candidates for developing effective and novel pharmaceutical agents. Additionally, this research direction should be further investigated by medicinal and organic chemists. These metabolites and plant extracts have been assessed for various bioactivities. Some metabolites demonstrated potent cytotoxic capacities (e.g., **11**, **13**, **19**, **23**, **24**, **47**, **53**, **57**, and **60**) similar or more than positive controls. Furthermore, the main reported mechanisms of their cytotoxic capacity included the induction of autophagy and apoptosis. These studies suggested that the plant and its sesquiterpenes could serve as promising chemotherapeutic agents for various cancer types. Additionally, compound **31** exhibited marked antiH1N1 potential. However, future in vitro and in vivo studies for chemo-preventive effects are essential to confirm this efficacy and to identify the compounds responsible for this chemo-preventive potential. Moreover, studies on the structure–activity relationships and/or derivatization of these metabolites should be carried out.

The anti-inflammatory potential of the plant/metabolites could support its usage as an alternative medicine for relieving and retarding inflammatory responses. Based on its powerful antioxidant and α-glucosidase inhibitory capacities, the plant could have remarkable potential for diabetes treatment. However, further studies for separating the active phytoconstituents accountable for these activities along with in vivo experiments and mechanistic studies are needed. Most of the reported activities of the plants have been attributed to its sesquiterpene contents. *C. abrotanoides* extracts and some metabolites have been established to display insecticidal capacity versus various pest species verifying their potential use in developing botanical insecticides. Comparing the results of biological studies with the plant traditional uses, it is obvious that only a few of the traditional uses have been scientifically verified. Moreover, the study of bioactivities of aqueous extracts is substantially needed for confirming the data of traditional use of decoctions and infusions. Some of the reported constituents are either not tested or possessed no notable effectiveness in the evaluated activities; therefore, an in silico screening for other potential bioactivities could be a clearly goal for future research studies. Most of the natural metabolites are frequently produced by the plants in small amounts; therefore, securing sufficient quantities of them for further clinical applications has become a persisting concern [83]. The technological progress, including genome sequencing and development of molecular biology and genetic techniques engineer the biosynthesis of many valuable natural metabolites [84]. In this review, highlighting the biosynthesis of these metabolites could draw the attention of molecular biologist- and genetic-interested researchers for isolating the genes accountable for the biosynthesis of these interesting metabolites and discovering the detailed mechanisms of their formation by various enzymes. This could allow the preparation of these metabolites and their analogs by engineering their biosynthetic pathways. Although the reported studies of *C. abrotanoides* revealed great promise, most of its activities have only been assessed in vitro. Accordingly, it is of the uttermost importance that the investigation of *C. abrotanoides* extracts and its constituents’ safety and efficacy testing should be carried out in in vivo studies. Lastly, clinical trials must be implemented to prove if the prominent activities of *C. abrotanoides* can result in clinical usability in an effective and safe manner and to completely certify its renowned use in the traditional medicines of various countries.

## Figures and Tables

**Table 1:** List of reported metabolites from *Carpesium abrotanoides*.

Compound Name	Plant Part	Extract/Fraction	Mol. Wt	Mol. Formula	City, Country	Ref.
Monoterpenes						
2,5-Dihydroxy-*p*-menthane (**1**)	Fruits	PE/acetone fraction of MeOH extract	172	C_10_H_20_O_2_	Tianquan, Sichuan, China	[44]
9-Hydroxy-thymol (**2**)	Whole plant	PE fraction of 95% EtOH extract	166	C_10_H_14_O_2_	China	[45]
8-Hydroxy-9,10-diisobutyryloxy-thymol (**3**)	Whole plant	PE fraction of 95% EtOH extract	338	C_18_H_26_O_6_	China	[45]
8,10-Dihydroxy-9-isobutyryloxy-thymol (**4**)	Whole plant	PE fraction of 95% EtOH extract	268	C_14_H_20_O_5_	China	[45]
10-Hydroxy-8,9-dioxy-isopropylidene-thymol (**5**)	Whole plant	PE fraction of 95% EtOH extract	238	C_13_H_18_O_4_	China	[45]
8-Hydroxy-9,10-dioxy-isopropylidene-thymol (**6**)	Whole plant	PE fraction of 95% EtOH extract	238	C_13_H_18_O_4_	China	[45]
**Guaianolide sesquiterpenes**						
Carpesia lactone (**7**)	Ripe seeds	Ether extract	248	C_15_H_20_O_3_	Japan	[46,47,48,49,50]
Tetrahydro-carpesia lactone (**8**)	Ripe seeds	Ether extract	252	C_15_H_24_O_3_	Japan	[46]
Dihydro-carpesia lactone (**9**)	Ripe seeds	Ether extract	250	C_15_H_22_O_3_	Japan	[48]
Inuviscolide (**10**)	Whole plant	EtOAc fraction of 95% EtOH extract	248	C_15_H_20_O_3_	Changyang, Hubei, China	[51]
	Whole plant	EtOAc fraction of 95% MeOH extract	-	-	Bozhou, Anhui, China	[52]
4-*Epi*-isoinuviscolide (**11**)	Aerial parts	PE/acetone fraction of 95% EtOH extract	248	C_15_H_20_O_3_	Tiger Leaping Gorge, Yunnan, China	[13]
8-*Epi*-isoinuviscolide (**12**)	Whole plant	EtOAc fraction of 95% EtOH extract	248	C_15_H_20_O_3_	Changyang, Hubei, China	[51]
4β,10β-dihydroxy-5αH-1,11(13)-guaidien-8α,12-olide (**13**)	Whole plant	EtOAc fraction of 95% EtOH extract	264	C_15_H_20_O_4_	Hebei, China	[53]
4*α*,5*α*-Epoxy-10*α*,14-dihydro-inuviscolide (**14**)	Aerial parts	CHCl_3_-soluble fraction of MeOH extract	248	C_15_H_20_O_3_	Korea	[51]
9β-Hydroxy-1βH,11αH-guaia-4,10(14)-dien-12,8α-olide (**15**)	Fruits	PE/acetone fraction of MeOH extract	248	C_15_H_20_O_3_	Tianquan, Sichuan, China	[44]
9β-Hydroxy-1βH,11βH-guaia-4,10(14)-dien-12,8α-olide (**16**)	Fruits	PE/acetone fraction of MeOH extract	248	C_15_H_20_O_3_	Tianquan, Sichuan, China	[44]
Caroguaianolide A (**17**)	Whole plant	EtOAc fraction of 95% EtOH extract	262	C_15_H_18_O_4_	Changyang, Hubei, China	[51]
Caroguaianolide B (**18**)	Whole plant	EtOAc fraction of 95% EtOH extract	280	C_15_H_20_O_5_	Changyang, Hubei, China	[51]
Caroguaianolide C (**19**)	Whole plant	EtOAc fraction of 95% EtOH extract	280	C_15_H_20_O_5_	Changyang, Hubei, China	[51]
Caroguaianolide D (**20**)	Whole plant	EtOAc fraction of 95% EtOH extract	280	C_15_H_20_O_5_	Changyang, Hubei, China	[51]
Caroguaianolide E (**21**)	Whole plant	EtOAc fraction of 95% EtOH extract	280	C_15_H_20_O_5_	Changyang, Hubei, China	[51]
Akihalin (**22**)	Whole plant	EtOAc fraction of 95% EtOH extract	280	C_15_H_20_O_5_	Changyang, Hubei, China	[51]
4β-Hydroxy,10β-hydroperoxyl,5αH,7αH,8βH-guaia-1,11(13)- dien-8α,12-olide (**23**)	Whole plant	EtOAc fraction of 95% EtOH extract	280	C_15_H_20_O_5_	Changyang, Hubei, China	[51]
4α-Hydroxy-9β,10β-epoxy-1βH,5αH-guaia-11(13)-en-8α,12-olide (**24**)	Whole plant	EtOAc fraction of 95% EtOH extract	264	C_15_H_20_O_4_	Changyang, Hubei, China	[51]
4α-Hydroxy-1βH-guaia-9,11(13)-dien-12,8α-olide (**25**)	Whole plant	EtOAc fraction of 95% EtOH extract	248	C_15_H_20_O_3_	Changyang, Hubei, China	[51]
1α,4α-Dihydroxy-guaia-11(13)-ene-12,8α-olide (**26**)	Whole plant	EtOAc fraction of 70% EtOH extract	266	C_15_H_22_O_4_	Changsha, Hunan, China	[54]
4α,5α-Dihydroxy-guaia-11(13)-en-12,8α-lactone (**27**)	Whole plant	EtOAc fraction of 70% EtOH extract	266	C_15_H_22_O_4_	Changsha, Hunan, China	[54]
Pseudo-guaianolide sesquiterpenes						
Carpesiolin (**28**)	Whole plant	CHCl_3_-soluble fraction of MeOH extract	264	C_15_H_20_O_4_	Japan	[55]
	Aerial parts	CHCl_3_-soluble fraction of MeOH extract	-	-	Korea	[51]
2,3-Dihydroaromomaticin (**29**)	Aerial parts	CHCl_3_-soluble fraction of MeOH extract	250	C_15_H_22_O_3_	Korea	[51]
	Whole plant	PE fraction of 95% MeOH extract	-	-	Bozhou, Anhui, China	[52]
Carabrolactone B (**30**)	Aerial parts	PE/acetone fraction of 95% EtOH extract	266	C_15_H_22_O_4_	Tiger Leaping Gorge, Yunnan, China	[13]
	Whole plant	EtOAc fraction of 95% EtOH extract	-	-	Changyang, Hubei, China	[51]
	Whole plant	EtOAc fraction of 95% MeOH extract	-	-	Bozhou, Anhui, China	[52]
2-Desoxy-4-*epi*-pulchellin (**31**)	Aerial parts	PE/acetone fraction of 95% EtOH extract	250	C_15_H_22_O_3_	Tiger Leaping Gorge, Yunnan, China	[13]
	Whole plant	EtOAc fraction of 95% EtOH extract	-	-	Changsha County of Hunan, China	[56]
	Whole plant	EtOAc fraction of 95% EtOH extract	-	-	Changyang, Hubei, China	[51]
	Whole plant	EtOAc fraction of 95% EtOH extract	-	-	Hebei, China	[53]
	Whole plant	EtOAc fraction of 95% MeOH extract	-	-	Bozhou, Anhui, China	[52]
3a*R*,4a*S*,5*S*,7a*S*,8*S*,9a*R*)-5-Hydroxy-4a,8-dimethyl-3-methylen-decahydroa-zuleno [6,5-*b*]furan-2(3*H*)-on (**32**)	Whole plant	EtOAc fraction of 95% EtOH extract	250	C_15_H_22_O_3_	Changyang, Hubei, China	[51]
Eudesmanolide sesquiterpenes						
Granilin (**33**)	Whole plant	CHCl_3_-soluble fraction of MeOH extract	264	C_15_H_20_O_4_	Japan	[57]
1.3-*Epi*-granilin (**34**)	Whole plant	EtOAc fraction of 95% MeOH extract	264	C_15_H_20_O_4_	Bozhou, Anhui, China	[52]
	Fruits	PE/acetone fraction of MeOH extract	-	-	Tianquan, Sichuan, China	[44]
Telekin (**35**)	Aerial parts	CHCl_3_-soluble fraction of MeOH extract	248	C_15_H_20_O_3_	Korea	[51]
	Whole plant	EtOAc fraction of 70% EtOH extract	-	-	Dao County, Hunan, China	[16]
	Whole plant	PE fraction of 95% MeOH extract	-	-	Bozhou, Anhui, China	[52]
Isotelekin (**36**)	Whole plant	EtOAc fraction of 95% MeOH extract	248	C_15_H_20_O_3_	Bozhou, Anhui, China	[52]
4(15)-β-Epoxyisotelekin (**37**)	Whole plant	EtOAc fraction of 95% EtOH extract	264	C_15_H_20_O_4_	Hebei, China	[53]
	Whole plant	EtOAc fraction of 95% MeOH extract	-	-	Bozhou, Anhui, China	[52]
5α-Epoxyalantolactone (**38**)	Whole plant	EtOAc fraction of 95% MeOH extract	248	C_15_H_20_O_3_	Bozhou, Anhui, China	[52]
Atractylenolide III (**39**)	Whole plant	EtOAc fraction of 95% MeOH extract	248	C_15_H_20_O_3_	Bozhou, Anhui, China	[52]
Ivalin (**40**)	Aerial parts	CHCl_3_-soluble fraction of MeOH extract	248	C_15_H_20_O_3_	Korea	[51]
	Whole plant	EtOAc fraction of 95% MeOH extract	-	-	Bozhou, Anhui, China	[52]
2α,5α-Dihydroxy-11αH-eudesma-4(15)-en-12,8β-olide (**41**)	Whole plant	EtOAc fraction of 70% EtOH extract	266	C_15_H_22_O_4_	Changsha, Hunan, China	[54]
	Whole plant	EtOAc fraction of 70% EtOH extract	-	-	Dao County, Hunan, China	[16]
(5α)-5-Hydroxyasperilin (**42**)	Whole plant	EtOAc fraction of 70% EtOH extract	266	C_15_H_22_O_4_	Changsha, Hunan, China	[54]
	Whole plant	PE fraction of 95% MeOH extract	-	-	Bozhou, Anhui, China	[52]
Carpabrotalactone B (**43**)	Whole plant	EtOAc fraction of 70% EtOH extract	264	C_15_H_20_O_4_	Changsha, Hunan, China	[54]
Carpabrotalactone C (**44**)	Whole plant	EtOAc fraction of 70% EtOH extract	264	C_15_H_20_O_4_	Changsha, Hunan, China	[58]
Oxoeudesm-11(13)-eno-12,8α-lactone (**45**)	Whole plant	EtOAc fraction of 70% EtOH extract	248	C_15_H_20_O_3_	Dao County, Hunan, China	[16]
5α-Hydroxy-4α,15-epoxy-11αH-eudesman-12,8β-olide (**46**)	Whole plant	EtOAc fraction of 95% EtOH extract	266	C_15_H_22_O_4_	Hebei, China	[53]
Germacranolide sesquiterpenes						
Ivaxillin (**47**)	Whole plant	CHCl_3_ fraction of MeOH extract	266	C_15_H_22_O_4_	Japan	[59]
	Aerial parts	PE/acetone fraction of 95% EtOH extract	-	-	Tiger Leaping Gorge, Yunnan, China	[13]
	Fruits	PE/acetone fraction of MeOH extract	-	-	Tianquan, Sichuan, China	[44]
11(13)-Dehydroivaxillin (**48**)	Whole plant	CHCl_3_ fraction of MeOH extract	264	C_15_H_20_O_4_	Japan	[59]
	Whole plant	EtOAc fraction of 95% EtOH extract	-	-	Changsha, Hunan, China	[56]
	Whole plant	EtOAc fraction of 70% EtOH extract	-	-	Changsha, Hunan, China	[54]
	Whole plant	PE fraction of 95% MeOH extract	-	-	Bozhou, Anhui, China	[52]
11,13-Didehydroivaxillin (**49**)	Aerial parts	CHCl_3_ fraction of MeOH extract	264	C_15_H_20_O_4_	Korea	[51]
Eriolin (**50**)	Whole plant	CHCl_3_-soluble fraction of MeOH extract	266	C_15_H_22_O_4_	Japan	[59]
	Aerial parts	PE/acetone fraction of 95% EtOH extract	-	-	Tiger Leaping Gorge, Yunnan, China	[13]
	Fruits	PE/acetone fraction of MeOH extract	-	-	Tianquan, Sichuan, China	[44]
	Whole plant	EtOAc fraction of 70% EtOH extract	-	-	Changsha, Hunan, China	[54]
Carabrolactone A (**51**)	Aerial parts	PE/acetone fraction of 95% EtOH extract	282	C_15_H_22_O_5_	Tiger Leaping Gorge, Yunnan, China	[13]
Carpabrotalactone A (**52**)	Whole plant	EtOAc fraction of 70% EtOH extract	296	C_16_H_24_O_5_	Changsha, Hunan, China	[54]
Eremophilanoide sesquiterpenes						
Carperemophilane A (**53**)	Whole plant	EtOAc fraction of 95% EtOH extract	268	C_15_H_24_O_4_	Changyang, Hubei, China	[60]
Carperemophilane B (**54**)	Whole plant	EtOAc fraction of 95% EtOH extract	268	C_15_H_24_O_4_	Changyang, Hubei, China	[60]
Sesquiterpenes with cyclopropane ring						
Carabrone (**55**)	Fruits	Ether extract	248	C_15_H_20_O_3_	Japan	[61]
	Whole plant	CHCl_3_-soluble fraction of MeOH extract	-	-	Japan	[59]
	Aerial parts	PE/acetone fraction of 95% EtOH extract	-	-	Tiger Leaping Gorge, Yunnan, China	[13]
	Aerial parts	CHCl_3_ fraction of MeOH extract	-	-	Korea	[51]
	Fruits	PE/acetone fraction of MeOH extract	-	-	Tianquan, Sichuan, China	[44]
	Whole plant	EtOAc fraction of 95% EtOH extract	-	-	Hebei, China	[53]
Carabrol (**56**)	Whole plant	CHCl_3_-soluble fraction of MeOH extract	250	C_15_H_22_O_3_	Japan	[59]
	Aerial parts	CHCl_3_-soluble fraction of MeOH extract	-	-	Korea	[51]
	Whole plant	EtOAc fraction of 95% EtOH extract	-	-	Changsha, Hunan, China	[56]
	Whole plant	EtOAc fraction of 95% EtOH extract	-	-	Hebei, China	[53]
	Whole plant	EtOAc fraction of 95% EtOH extract	-	-	Changyang, Hubei, China	[60]
	Whole plant	EtOAc fraction of 95% MeOH extract	-	-	Bozhou, Anhui, China	[52]
11*R*-Hydroxycarabrol (**57**)	Whole plant	CHCl_3_-soluble fraction of 95% EtOH	268	C_15_H_24_O_4_	Henan, China	[62]
Carabrol-4-O-palmitate (**58**)	Whole plant	EtOAc fraction of 95% EtOH extract	488	C_31_H_52_O_4_	Hebei, China	[53]
Carabrol-4-O-linoleate (**59**)	Whole plant	EtOAc fraction of 95% EtOH extract	516	C_33_H_56_O_4_	Hebei, China	[53]
Dimeric sesquiterpenes						
Dicarabrol (**60**)	Whole plant	EtOAc fraction of EtOH extract	500	C_30_H_44_O_6_	Changsha, Hunan, China	[56]
Dicarabrol A (**61**)	Whole plant	CH_2_Cl_2_-soluble fraction of 95% EtOH extract	516	C_30_H_44_O_7_	Henan, China	[63]
Dicarabrol B (**62**)	Whole plant	CHCl_3_-soluble fraction of 95% EtOH	500	C_30_H_44_O_6_	Henan, China	[62]
Dicarabrol C (**63**)	Whole plant	CHCl_3_-soluble fraction of 95% EtOH	500	C_30_H_44_O_6_	Henan, China	[62]
Dicarabrone A (**64**)	Whole plant	CHCl_3_-soluble fraction of 95% EtOH extract	496	C_30_H_40_O_6_	Henan, China	[64]
Dicarabrone B (**65**)	Whole plant	CHCl_3_-soluble fraction of 95% EtOH extract	496	C_30_H_40_O_6_	Henan, China	[64]
Dicarabrone C (**66**)	Whole plant	CH_2_Cl_2_-soluble fraction of 95% EtOH extract	512	C_30_H_40_O_7_	Henan, China	[63]
Carabrodilactone A (**67**)	Whole plant	EtOAc fraction of 95% EtOH extract	540	C_32_H_44_O_7_	Pu’an, GuiZhou, China	[65]
Carabrodilactone B (**68**)	Whole plant	EtOAc fraction of 95% EtOH extract	542	C_32_H_46_O_7_	Pu’an, GuiZhou, China	[65]
Carabrodilactone C (**69**)	Whole plant	EtOAc fraction of 95% EtOH extract	556	C_32_H_44_O_8_	Pu’an, GuiZhou, China	[65]
Carabrodilactone D (**70**)	Whole plant	EtOAc fraction of 95% EtOH extract	556	C_32_H_44_O_8_	Pu’an, GuiZhou, China	[65]
Carabrodilactone E (**71**)	Whole plant	EtOAc fraction of 95% EtOH extract	556	C_32_H_44_O_8_	Pu’an, GuiZhou, China	[65]
Carpedilactone A (**72**)	Whole plant	EtOAc fraction of 95% EtOH extract	494	C_30_H_38_O_6_	Pu’an, GuiZhou, China	[65]
Carpedilactone B (**73**)	Whole plant	EtOAc fraction of 95% EtOH extract	494	C_30_H_38_O_6_	Pu’an, GuiZhou, China	[65]
Dipulchellin A (**74**)	Whole plant	CH_2_Cl_2_-soluble fraction of 95% EtOH extract	500	C_30_H_44_O_6_	Henan, China	[63]
Faberidilactone A (**75**)	Whole plant	EtOAc fraction of 95% EtOH extract	478	C_30_H_38_O_5_	Pu’an, GuiZhou, China	[65]
Faberidilactone C (**76**)	Whole plant	EtOAc fraction of 95% EtOH extract	478	C_30_H_38_O_5_	Pu’an, GuiZhou, China	[65]
Other metabolites						
(3*R*,6*R*,7*E*)-3-Hydroxy-4,7-megastigmadien-9-one (**77**)	Whole plant	PE fraction of 95% EtOH extract	208	C_13_H_20_O_2_	China	[45]
Blumenol A (**78**)	Whole plant	PE fraction of 95% EtOH extract	224	C_13_H_20_O_3_	China	[45]
4-(3-Oxobut-l-enylidene)-3,5,5-trimethylcyclohex-2-en-l-one (**79**)	Whole plant	PE fraction of 95% EtOH extract	208	C_13_H_16_O_2_	China	[45]
Loliolide (**80**)	Whole plant	PE fraction of 95% EtOH extract	196	C_11_H_16_O_3_	China	[45]
Methyl 8-[2(5*H*)-furanon-5-yl]octanoate (**81**)	Whole plant	EtOAc fraction of 70% EtOH extract	240	C_13_H_20_O_4_	Changsha, Hunan, China	[58]
Carpesiumaleimide A (**82**)	Whole plant	EtOAc fraction of 95% EtOH extract	253	C_13_H_19_NO_4_	Changyang, Hubei, China	[60]
Carpesiumaleimide C (**83**)	Whole plant	EtOAc fraction of 95% EtOH extract	267	C_14_H_21_NO_4_	Changyang, Hubei, China	[60]
Carpesiumaleimide B (**84**)	Whole plant	EtOAc fraction of 95% EtOH extract	267	C_14_H_21_NO_4_	Changyang, Hubei, China	[60]
Triacontane (**85**)	Ripe seeds	Ether extract	422	C_30_H_62_	Japan	[46]
Ceryl alcohol (**86**)	Ripe seeds	Ether extract	382	C_26_H_54_O	Japan	[66]
12,13-Epoxylinolenic acid (**87**)	Roots	PE fraction of 80% EtOH extract	294	C_18_H_30_O_3_	Fuyang, Zhejinag, China	[17]
(5E,10E)-6,10,14-trimethyl-pentadeca-5,10,13-trience-2,12-dione (**88**)	Roots	PE fraction of 80% EtOH extract	276	C_18_H_28_O_2_	Fuyang, Zhejinag, China	[17]
5α-hydroxy-7αH,11αH-eudesm-4(15)-en-12,8β-lactone (**90**)	Roots	PE fraction of 80% EtOH extract	250	C_15_H_22_O_3_	Fuyang, Zhejinag, China	[17]
Palmitic acid (**91**)	Roots	PE fraction of 80% EtOH extract	256	C_16_H_32_O_2_	Fuyang, Zhejinag, China	[17]
β-Bisabolene (**92**)	Whole plant	Essential oil	204	C_15_H_24_	Japan	[67]
	Whole plant	Essential oil	-	-	Hubei, China	[15]
Dihydroactinidiolide (**93**)	Whole plant	Essential oil	182	C_11_H_18_	Japan	[67]
γ-Nonalactone (**94**)	Whole plant	Essential oil	156	C_9_H_16_O_2_	Japan	[67]
Butylphthalide (**95**)	Whole plant	Essential oil	190	C_9_H_16_O_2_	Japan	[67]
Eudesma-5,11(13)-dien-8,12-olide (**96**)	Whole plant	Essential oil	232	C_15_H_20_O_2_	Hubei, China	[15]
Caryophyllene oxide (**97**)	Whole plant	Essential oil	220	C_15_H_24_O	Hubei, China	[15]
	Aerial parts	Essential oil			Abbottabad, Pakistan	[32]
3α,4α-4-Methylcholesta-8,24-dien-3-ol (**98**)	Whole plant	Essential oil	398	C_28_H_46_O	Hubei, China	[15]
Phytol (**99**)	Whole plant	Essential oil	296	C_20_H_40_O	Hubei, China	[15]
2-(E)-Decenal (**100**)	Whole plant	Essential oil	154	C_10_H_18_O	Hubei, China	[15]
Heptacosane (**101**)	Whole plant	Essential oil	380	C_27_H_56_	Hubei, China	[15]
Eicosane (**102**)	Whole plant	Essential oil	280	C_20_H_42_	Hubei, China	[15]
Tetracosane (**103**)	Whole plant	Essential oil	338	C_24_H_50_	Hubei, China	[15]
4-(1-Methylethyl)-benzaldehyde (**104**)	Whole plant	Essential oil	148	C_10_H_12_O	Hubei, China	[15]
2,6,10-Trimethyl-dodecane (**105**)	Whole plant	Essential oil	212	β-cedrene	Hubei, China	[15]
Torreyol (**106**)	Whole plant	Essential oil	222	C_15_H_26_O	Hubei, China	[15]
Androst-5,7-dien-3-ol-17-one (**107**)	Whole plant	Essential oil	286	C_19_H_26_O_2_	Hubei, China	[15]
β-Cedrene (**108**)	Whole plant	Essential oil	204	C_15_H_24_	Hubei, China	[15]
α-Curcumene (**109**)	Whole plant	Essential oil	202	C_15_H_22_	Hubei, China	[15]
2,6,10-Trimethyl-tetradecane (**110**)	Whole plant	Essential oil	240	C_17_H_36_	Hubei, China	[15]
Caryophyllene (**111**)	Aerial parts	Essential oil	204	C_15_H_24_	Abbottabad, Pakistan	[32]
Trans-nerolidol (**112**)	Aerial parts	Essential oil	222	C_15_H_26_O	Abbottabad, Pakistan	[32]
Geranyl isobutyrate (**113**)	Aerial parts	Essential oil	224	C_14_H_24_O_2_	Abbottabad, Pakistan	[32]
δ-cadinene (**114**)	Aerial parts	Essential oil	204	C_15_H_24_	Abbottabad, Pakistan	[32]
β-Eudesmene (**115**)	Aerial parts	Essential oil	204	C_15_H_24_	Abbottabad, Pakistan	[32]
Trans-lachnophyllum ester (**116**)	Aerial parts	Essential oil	176	C_11_H_12_O_2_	Abbottabad, Pakistan	[32]
α-Caryophyllene (**117**)	Aerial parts	Essential oil	204	C_15_H_24_	Abbottabad, Pakistan	[32]
Neryl 3-methylbutyrate (**118**)	Aerial parts	Essential oil	238	C_15_H_26_O_2_	Abbottabad, Pakistan	[32]

**Table 2:** Biological activity of reported metabolites from *Carpesium abrotanoides*.

Compound Name	Biological Activity	Assay/Organism/ Cell Line	Biological Results		Ref.
			Compound	Positive Control	
Inuviscolide (**10**)	Cytotoxicity	MTT/MDA-MB-231	17.26 μM (IC_50_)	Mitomycin C 4.56 μM (IC_50_)	[51]
8-*Epi*-isoinuviscolide (**12**)	Cytotoxicity	MTT/MDA-MB-231	16.87 μM (IC_50_)	Mitomycin C 4.56 μM (IC_50_)	[51]
4*α*,5*α*-Epoxy-10*α*,14-dihydro-inuviscolide (**14**)	Cytotoxicity	SRB/L1210	1.8 μM (ED_50_)	Cisplatin 0.07 μM (ED_50_)	[51]
		SRB/A549	5.5 μM (ED_50_)	Cisplatin 4.1 μM (ED_50_)	[51]
		SRB/SK-OV-3	3.4 μM (ED_50_)	Cisplatin 2.8 μM (ED_50_)	[51]
		SRB/SK-MEL-2	3.3 μM (ED_50_)	Cisplatin 2.6 μM (ED_50_)	[51]
		SRB/XF-498	4.4 μM (ED_50_)	Cisplatin 2.9 μM (ED_50_)	[51]
		SRB/HCT-15	5.8 μM (ED_50_)	Cisplatin 7.1 μM (ED_50_)	[51]
Caroguaianolide A (**17**)	Cytotoxicity	MTT/MDA-MB-231	7.96 μM (IC_50_)	Mitomycin C 4.56 μM (IC_50_)	[51]
		MTT/HGC-27	10.47 μM (IC_50_)	Mitomycin C 6.68 μM (IC_50_)	[51]
Caroguaianolide B (**18**)	Cytotoxicity	MTT/MDA-MB-231	4.25 μM (IC_50_)	Mitomycin C 4.56 μM (IC_50_)	[51]
		MTT/HGC-27	6.47 μM (IC_50_)	Mitomycin C 6.68 μM (IC_50_)	[51]
Caroguaianolide C (**19**)	Cytotoxicity	MTT/MDA-MB-231	2.67 μM (IC_50_)	Mitomycin C 4.56 μM (IC_50_)	[51]
		MTT/HGC-27	4.83 μM (IC_50_)	Mitomycin C 6.68 μM (IC_50_)	[51]
Caroguaianolide D (**20**)	Cytotoxicity	MTT/MDA-MB-231	17.21 μM (IC_50_)	Mitomycin C 4.56 μM (IC_50_)	[51]
Caroguaianolide E (**21**)	Cytotoxicity	MTT/MDA-MB-231	18.37 μM (IC_50_)	Mitomycin C 4.56 μM (IC_50_)	[51]
Akihalin (**22**)	Cytotoxicity	MTT/MDA-MB-231	4.83 μM (IC_50_)	Mitomycin C 4.56 μM (IC_50_)	[51]
		MTT/HGC-27	7.35 μM (IC_50_)	Mitomycin C 6.68 μM (IC_50_)	[51]
4β-Hydroxy,10β-hydroperoxyl,5αH,7αH,8βH-guaia-1,11(13)- dien-8α,12-olide (**23**)	Cytotoxicity	MTT/MDA-MB-231	5.79 μM (IC_50_)	Mitomycin C 4.56 μM (IC_50_)	[51]
		MTT/HGC-27	12.34 μM (IC_50_)	Mitomycin C 6.68 μM (IC_50_)	[51]
4α-Hydroxy-1βH-guaia-9,11(13)-dien-12,8α-olide (**25**)	Cytotoxicity	MTT/MDA-MB-231	4.07 μM (IC_50_)	Mitomycin C 4.56 μM (IC_50_)	[51]
		MTT/HGC-27	8.95 μM (IC_50_)	Mitomycin C 6.68 μM (IC_50_)	[51]
1α,4α-Dihydroxy-guaia-11(13)-ene-12,8α-olide (**26**)	Cytotoxicity	CCK-8/HeLa	15.6 μM (IC_50_)	Cisplatin 11.1 μM (IC_50_)	[54]
		CCK-8/Caco-2	28.5 μM (IC_50_)	Cisplatin 22.1 μM (IC_50_)	[54]
	Anti-influenza A	CCK-8/H1N1	1.3 μM (IC_50_)	Oseltamivir 0.05 μM (IC_50_)	[54]
4α,5α-Dihydroxy-guaia-11(13)-en-12,8α-lactone (**27**)	Cytotoxicity	CCK-8/ISK	30.3 μM (IC_50_)	Cisplatin 31.2 μM (IC_50_)	[54]
		CCK-8/HeLa	9.9 μM (IC_50_)	Cisplatin 11.1 μM (IC_50_)	[54]
		CCK-8/A549	47.4 μM (IC_50_)	Cisplatin 40.1 μM (IC_50_)	[54]
		CCK-8/Caco-2	16.4 μM (IC_50_)	Cisplatin 22.1 μM (IC_50_)	[54]
	Anti-influenza A	CCK-8/H1N1	0.4 μM (IC_50_)	Oseltamivir 0.05 μM (IC_50_)	[54]
Carpesiolin (**28**)	Cytotoxicity	SRB/L1210	9.6 μM (ED_50_)	Cisplatin 0.07 μM (ED_50_)	[51]
		SRB/A549	13.2 μM (ED_50_)	Cisplatin 4.1 μM (ED_50_)	[51]
		SRB/SK-OV-3	9.8 μM (ED_50_)	Cisplatin 2.8 μM (ED_50_)	[51]
		SRB/SK-MEL-2	7.7 μM (ED_50_)	Cisplatin 2.6 μM (ED_50_)	[51]
		SRB/XF-498	9.3 μM (ED_50_)	Cisplatin 2.9 μM (ED_50_)	[51]
		SRB/HCT-15	6.7 μM (ED_50_)	Cisplatin 7.1 μM (ED_50_)	[51]
2,3-Dihydroaromomaticin (**29**)	Cytotoxicity	SRB/L1210	5.0 μM (ED_50_)	Cisplatin 0.07 μM (ED_50_)	[51]
		SRB/A549	7.1 μM (ED_50_)	Cisplatin 4.1 μM (ED_50_)	[51]
		SRB/SK-OV-3	3.9 μM (ED_50_)	Cisplatin 2.8 μM (ED_50_)	[51]
		SRB/SK-MEL-2	4.0 μM (ED_50_)	Cisplatin 2.6 μM (ED_50_)	[51]
		SRB/XF-498	3.3 μM (ED_50_)	Cisplatin 2.9 μM (ED_50_)	[51]
		SRB/HCT-15	8.1 μM (ED_50_)	Cisplatin 7.1 μM (ED_50_)	[51]
		MTT/A549	7.09 μM (IC_50_)	Vinblastine 18.5 μM (IC_50_)	[52]
		MTT/HepG2	9.03 μM (IC_50_)	Vinblastine 9.29 μM (IC_50_)	[52]
		MTT/MDA-MB-231	5.16 μM (IC_50_)	Vinblastine 42.51 μM (IC_50_)	[52]
		MTT/HCT116	10.15 μM (IC_50_)	Vinblastine 30.62 μM (IC_50_)	[52]
		MTT/CNE2	12.99 μM (IC_50_)	Vinblastine 1.16 μM (IC_50_)	[52]
2-Desoxy-4-*epi*-pulchellin (**31**)	Cytotoxicity	CCK-8/K562	17.0 μM (IC_50_)	Taxol 3.8 μM (IC_50_)	[56]
		CCK-8/MCF-7	11.0 μM (IC_50_)	Taxol 2.3 μM (IC_50_)	[56]
		CCK-8/HeLa	6.0 μM (IC_50_)	Taxol 2.9 μM (IC_50_)	[56]
		CCK-8/DU145	3.1 μM (IC_50_)	Taxol 3.1 μM (IC_50_)	[56]
		CCK-8/U937	2.2 μM (IC_50_)	Taxol 2.1 μM (IC_50_)	[56]
		CCK-8/H1975	13.0 μM (IC_50_)	Taxol 2.8 μM (IC_50_)	[56]
		CCK-8/SGC-7901	22.0 μM (IC_50_)	Taxol 9.8 μM (IC_50_)	[56]
		CCK-8/A549	6.2 μM (IC_50_)	Taxol 2.1 μM (IC_50_)	[56]
		CCK-8/MOLT-4	5.5 μM (IC_50_)	Taxol 2.7 μM (IC_50_)	[56]
		CCK-8/HL60	2.4 μM (IC_50_)	Taxol 3.8 μM (IC_50_)	[56]
		MTT/MDA-MB-231	18.67 μM (IC_50_)	Mitomycin C 4.56 μM (IC_50_)	[51]
		MTT/A549	6.04 μM (IC_50_)	Vinblastine 18.5 μM (IC_50_)	[52]
		MTT/HepG2	18.25 μM (IC_50_)	Vinblastine 9.29 μM (IC_50_)	[52]
		MTT/MDA-MB-231	21.55 μM (IC_50_)	Vinblastine 42.51 μM (IC_50_)	[52]
	Antiviral	CPE inhibition/H1N1	29.3 μM (IC_50_)	Osehamivir 0.025 μM (IC_50_)	[56]
		CPE inhibition/H3N2	47.3 μM (IC_50_)	Osehamivir 0.015 μM (IC_50_)	[56]
	Antimycobacterial	GFPMA/*Mycobacterium tuberculosis* strains H37Rv, H37Ra	7.6 μM (MIC)	Isoniazid, 2.0 μM (MIC)	[56]
3a*R*,4a*S*,5*S*,7a*S*,8*S*,9a*R*)-5-Hydroxy-4a,8-dimethyl-3-methylen-decahydroa-zuleno [6,5-*b*]furan-2(3H)-on (**32**)	Cytotoxicity	MTT/MDA-MB-231	5.32 μM (IC_50_)	Mitomycin C 4.56 μM (IC_50_)	[51]
		MTT/HGC-27	11.44 μM (IC_50_)	Mitomycin C 6.68 μM (IC_50_)	[51]
Telekin (**35**)	Cytotoxicity	SRB/L1210	7.5 μM (ED_50_)	Cisplatin 0.07 μM ED_50_)	[51]
		SRB/A549	7.5 μM (ED_50_)	Cisplatin 4.1 μM (ED_50_)	[51]
		SRB/SK-OV-3	4.1 μM (ED_50_)	Cisplatin 2.8 μM (ED_50_)	[51]
		SRB/SK-MEL-2	4.9 μM (ED_50_)	Cisplatin 2.6 μM (ED_50_)	[51]
		SRB/XF-498	6.6 μM (ED_50_)	Cisplatin 2.9 μM (ED_50_)	[51]
		SRB/HCT-15	6.7 μM (ED_50_)	Cisplatin 7.1 μM (ED_50_)	[51]
		HepG2/CCK-8	2.95 μM (IC_50_)	Paclitaxel	[16]
		MTT/A549	11.08 μM (IC_50_)	Vinblastine 18.5 μM (IC_50_)	[52]
		MTT/HepG2	7.89 μM (IC_50_)	Vinblastine 9.29 μM (IC_50_)	[52]
		MTT/MDA-MB-231	9.46 μM (IC_50_)	Vinblastine 42.51 μM (IC_50_)	[52]
		MTT/HCT116	9.69 μM (IC_50_)	Vinblastine 30.62 μM (IC_50_)	[52]
		MTT/CNE2	13.66 μM (IC_50_)	Vinblastine 1.16 μM (IC_50_)	[52]
5*α*-Epoxyalantolactone (**38**)	Cytotoxicity	MTT/A549	3.51 μM (IC_50_)	Vinblastine 18.5 μM (IC_50_)	[52]
		MTT/HepG2	2.73 μM (IC_50_)	Vinblastine 9.29 μM (IC_50_)	[52]
		MTT/MDA-MB-231	4.18 μM (IC_50_)	Vinblastine 42.51 μM (IC_50_)	[52]
		MTT/HCT116	3.75 μM (IC_50_)	Vinblastine 30.62 μM (IC_50_)	[52]
		MTT/CNE2	7.21 μM (IC_50_)	Vinblastine 1.16 μM (IC_50_)	[52]
		MTT/NCM460	13.66 μM (IC_50_)	Vinblastine	[52]
Ivalin (**40**)	Cytotoxicity	SRB/L1210	7.5 μM (ED_50_)	Cisplatin 0.07 μM ED_50_)	[51]
		SRB/A549	9.9 μM (ED_50_)	Cisplatin 4.1 μM (ED_50_)	[51]
		SRB/SK-OV-3	3.5 μM (ED_50_)	Cisplatin 2.8 μM (ED_50_)	[51]
		SRB/SK-MEL-2	3.0 μM (ED_50_)	Cisplatin 2.6 μM (ED_50_)	[51]
		SRB/XF-498	2.9 μM (ED_50_)	Cisplatin 2.9 μM (ED_50_)	[51]
		SRB/HCT-15	2.6 μM (ED_50_)	Cisplatin 7.1 μM (ED_50_)	[51]
		MTT/A549	7.77 μM (IC_50_)	Vinblastine 18.5 μM (IC_50_)	[52]
		MTT/HepG2	6.1 μM (IC_50_)	Vinblastine 9.29 μM (IC_50_)	[52]
		MTT/MDA-MB-231	7.42 μM (IC_50_)	Vinblastine 42.51 μM (IC_50_)	[52]
		MTT/HCT116	13.12 μM (IC_50_)	Vinblastine 30.62 μM (IC_50_)	[52]
		MTT/CNE2	23.6 μM (IC_50_)	Vinblastine 1.16 μM (IC_50_)	[52]
2α,5α-Dihydroxy-11αH-eudesma-4(15)-en-12,8β-olide (**41**)	Cytotoxicity	CCK-8/ISK	21.8 μM (IC_50_)	Cisplatin 31.2 μM (IC_50_)	[54]
		CCK-8/HeLa	8.5 μM (IC_50_)	Cisplatin 11.1 μM (IC_50_)	[54]
		CCK-8/Sw620	42.3 μM (IC_50_)	Cisplatin 37.9 μM (IC_50_)	[54]
		CCK-8/RBE	45.1 μM (IC_50_)	Cisplatin 42.5 μM (IC_50_)	[54]
		CCK-8/Caco-2	42.6 μM (IC_50_)	Cisplatin 22.1 μM (IC_50_)	[54]
		CCK-8/HepG2	9.83 μM (IC_50_)	Paclitaxel	[16]
(5α)-5-Hydroxyasperilin (**42**)	Cytotoxicity	CCK-8/ISK	25.8 μM (IC_50_)	Cisplatin 31.2 μM (IC_50_)	[54]
		CCK-8/HeLa	10.7 μM (IC_50_)	Cisplatin 11.1 μM (IC_50_)	[54]
		CCK-8/Sw620	28.1 μM (IC_50_)	Cisplatin 37.9 μM (IC_50_)	[54]
		CCK-8/RBE	28.4 μM (IC_50_)	Cisplatin 42.5 μM (IC_50_)	[54]
		CCK-8/Caco-2	21.5 μM (IC_50_)	Cisplatin 22.1 μM (IC_50_)	[54]
Carpabrotalactone B (**43**)	Cytotoxicity	CCK-8/ISK	33.8 μM (IC_50_)	Cisplatin 31.2 μM (IC_50_)	[54]
		CCK-8/HeLa	25.6 μM (IC_50_)	Cisplatin 11.1 μM (IC_50_)	[54]
		CCK-8/Caco-2	45.4 μM (IC_50_)	Cisplatin 22.1 μM (IC_50_)	[54]
	Anti-influenza A	CCK-8/H1N1	6.2 μM (IC_50_)	Oseltamivir 0.05 μM (IC_50_)	[54]
Carpabrotalactone C (**44**)	Cytotoxicity	CCK-8/A549	19.1 μM (IC_50_)	Cisplatin 12.77 μM (IC_50_)	[58]
		CCK-8/SMMC-7721	23.68 μM (IC_50_)	Cisplatin 5.1 μM (IC_50_)	[58]
		CCK-8/MCF-7	20.32 μM (IC_50_)	Cisplatin 12.6 μM (IC_50_)	[58]
		CCK-8/SW480	11.46 μM (IC_50_)	Cisplatin 6.67 μM (IC_50_)	[58]
Oxoeudesm-11(13)-eno-12,8α-lactone (**45**)	Cytotoxicity	CCK-8/HepG2	4.15 μM (IC_50_)	Paclitaxel	[16]
	Anti-inflammatory	NO inhibition/LPS	63.23 μM (IC_50_)	Dexamethasone 3.61 μM (IC_50_)	[58]
11(13)-Dehydroivaxillin (**48**)	Cytotoxicity	CCK-8/K562	1.7 μM (IC_50_)	Taxol 3.8 μM (IC_50_)	[56]
		CCK-8/MCF-7	4.1 μM (IC_50_)	Taxol 2.3 μM (IC_50_)	[56]
		CCK-8/HeLa	1.0 μM (IC_50_)	Taxol 2.9 μM (IC_50_)	[56]
		CCK-8/DU145	0.38 μM (IC_50_)	Taxol 3.1 μM (IC_50_)	[56]
		CCK-8/U937	0.21 μM (IC_50_)	Taxol 2.1 μM (IC_50_)	[56]
		CCK-8/H1975	5.4 μM (IC_50_)	Taxol 2.8 μM (IC_50_)	[56]
		CCK-8/SGC-7901	6.4 μM (IC_50_)	Taxol 9.8 μM (IC_50_)	[56]
		CCK-8/A549	2.0 μM (IC_50_)	Taxol 2.1 μM (IC_50_)	[56]
		CCK-8/MOLT-4	1.2 μM (IC_50_)	Taxol 2.7 μM (IC_50_)	[56]
		CCK-8/ISK	13.7 μM (IC_50_)	Cisplatin 31.2 μM (IC_50_)	[54]
		CCK-8/HeLa	12.3 μM (IC_50_)	Cisplatin 11.1 μM (IC_50_)	[54]
		CCK-8/Sw620	12.1 μM (IC_50_)	Cisplatin 37.9 μM (IC_50_)	[54]
		CCK-8/RBE	7.8 μM (IC_50_)	Cisplatin 42.5 μM (IC_50_)	[54]
		CCK-8/Caco-2	20.4 μM (IC_50_)	Cisplatin 22.1 μM (IC_50_)	[54]
		MTT/A549	17.6 μM (IC_50_)	Vinblastine 18.5 μM (IC_50_)	[52]
		MTT/HepG2	14.59 μM (IC_50_)	Vinblastine 9.29 μM (IC_50_)	[52]
		MTT/MDA-MB-231	10.88 μM (IC_50_)	Vinblastine 42.51 μM (IC_50_)	[52]
		CCK-8/HL60	0.18 μM (IC_50_)	Taxol 3.8 μM (IC_50_)	[60]
	Anti-influenza A	CCK-8/H1N1	11.6 μM (IC_50_)	Oseltamivir 0.05 μM (IC_50_)	[54]
	Antiviral	CPE inhibition/H1N1	10.8 μM (IC_50_)	Osehamivir 0.025 μM (IC_50_)	[56]
		CPE inhibition/H3N2	11.6 μM (IC_50_)	Osehamivir 0.015 μM (IC_50_)	[56]
	Antimycobacterial	GFPMA/*Mycobacterium tuberculosis* strains H37Rv, H37Ra	6.0 μM (MIC)	Isoniazid, 2.0 μM (MIC)	[56]
11,13-Didehydroivaxillin (**49**)	Cytotoxicity	SRB/L1210	11.4 μM (ED_50_)	Cisplatin 0.07 μM ED_50_)	[51]
		SRB/A549	18.4 μM (ED_50_)	Cisplatin 4.1 μM (ED_50_)	[51]
		SRB/SK-OV-3	9.7 μM (ED_50_)	Cisplatin 2.8 μM (ED_50_)	[51]
		SRB/SK-MEL-2	9.5 μM (ED_50_)	Cisplatin 2.6 μM (ED_50_)	[51]
		SRB/XF-498	10.6 μM (ED_50_)	Cisplatin 2.9 μM (ED_50_)	[51]
		SRB/HCT-15	7.4 μM (ED_50_)	Cisplatin 7.1 μM (ED_50_)	[51]
Eriolin (**50**)	Cytotoxicity	CCK-8/ISK	41.6 μM (IC_50_)	Cisplatin 31.2 μM (IC_50_)	[54]
		CCK-8/HeLa	14.8 μM (IC_50_)	Cisplatin 11.1 μM (IC_50_)	[54]
		CCK-8/Sw620	46.4 μM (IC_50_)	Cisplatin 37.9 μM (IC_50_)	[54]
		CCK-8/Caco-2	12.7 μM (IC_50_)	Cisplatin 22.1 μM (IC_50_)	[54]
	Anti-influenza A	CCK-8/H1N1	16.4 μM (IC_50_)	Oseltamivir 0.05 μM (IC_50_)	[54]
Carpabrotalactone A (**52**)	Cytotoxicity	CCK-8/ISK	21.0 μM (IC_50_)	Cisplatin 31.2 μM (IC_50_)	[54]
		CCK-8/HeLa	9.6 μM (IC_50_)	Cisplatin 11.1 μM (IC_50_)	[54]
		CCK-8/Sw620	44.5 μM (IC_50_)	Cisplatin 37.9 μM (IC_50_)	[54]
		CCK-8/Caco-2	13.4 μM (IC_50_)	Cisplatin 22.1 μM (IC_50_)	[54]
Carperemophilane A (**53**)	Cytotoxicity	MTT/MDA-MB-231	22.67 μM (IC_50_)	Mitomycin C 4.89 μM (IC_50_)	[60]
		MTT/HGC-27	24.83 μM (IC_50_)	Mitomycin C 6.73 μM (IC_50_)	[60]
Carperemophilane B (**54**)	Cytotoxicity	MTT/MDA-MB-231	34.83 μM (IC_50_)	Mitomycin C 4.89 μM (IC_50_)	[60]
		MTT/HGC-27	37.35 μM (IC_50_)	Mitomycin C 6.73 μM (IC_50_)	[60]
Carabrone (**55**)	Antifungal	Spore germination/*Colletotrichum lagenarium*	7.1 μg/mL (EC_50_)	Chlorothalonil 0.75 μg/mL (EC_50_)	[69]
	Cytotoxicity	SRB/L1210	6.2 μM (ED_50_)	Cisplatin 0.07 μM ED_50_)	[51]
		SRB/A549	17.1 μM (ED_50_)	Cisplatin 4.1 μM (ED_50_)	[51]
		SRB/SK-OV-3	11.3 μM (ED_50_)	Cisplatin 2.8 μM (ED_50_)	[51]
		SRB/SK-MEL-2	8.5 μM (ED_50_)	Cisplatin 2.6 μM (ED_50_)	[51]
		SRB/XF-498	19.8 μM (ED_50_)	Cisplatin 2.9 μM (ED_50_)	[51]
		SRB/HCT-15	7.4 μM (ED_50_)	Cisplatin 7.1 μM (ED_50_)	[51]
		MTT/HCT117	17.13 μM (IC_50_)	Doxorubicin 0.20 μM (IC_50_)	[70]
		MTT/CCRF-CEM	43.66 μM (IC_50_)	Doxorubicin 0.003 μM (IC_50_)	[70]
		MTT/K562	19.42 μM (IC_50_)	Doxorubicin 0.04 μM (IC_50_)	[70]
		MTT/HL60	23.29 μM (IC_50_)	Doxorubicin < 0.001 μM (IC_50_)	[70]
Carabrol (**56**)	Cytotoxicity	SRB/L1210	11.4 μM (ED_50_)	Cisplatin 0.07 μM ED_50_)	[51]
		SRB/A549	13.1 μM (ED_50_)	Cisplatin 4.1 μM (ED_50_)	[51]
		SRB/SK-OV-3	10.6 μM (ED_50_)	Cisplatin 2.8 μM (ED_50_)	[51]
		SRB/SK-MEL-2	11.2 μM (ED_50_)	Cisplatin 2.6 μM (ED_50_)	[51]
		SRB/XF-498	13.5 μM (ED_50_)	Cisplatin 2.9 μM (ED_50_)	[51]
		SRB/HCT-15	10.8 μM (ED_50_)	Cisplatin 7.1 μM (ED_50_)	[51]
		CCK-8/K562	4.7 μM (IC_50_)	Taxol 3.8 μM (IC_50_)	[56]
		CCK-8/MCF-7	47.0 μM (IC_50_)	Taxol 2.3 μM (IC_50_)	[56]
		CCK-8/HeLa	6.6 μM (IC_50_)	Taxol 2.9 μM (IC_50_)	[56]
		CCK-8/DU145	2.6 μM (IC_50_)	Taxol 3.1 μM (IC_50_)	[56]
		CCK-8/U937	0.94 μM (IC_50_)	Taxol 2.1 μM (IC_50_)	[56]
		CCK-8/H1975	4.0 μM (IC_50_)	Taxol 2.8 μM (IC_50_)	[56]
		CCK-8/MOLT-4	8.9 μM (IC_50_)	Taxol 2.7 μM (IC_50_)	[56]
		CCK-8/HL60	0.36 μM (IC_50_)	Taxol 3.8 μM (IC_50_)	[56]
		MTT/MDA-MB-231	7.45 μM (IC_50_)	Mitomycin C 4.89 μM (IC_50_)	[60]
		MTT/HGC-27	10.27 μM (IC_50_)	Mitomycin C 6.73 μM (IC_50_)	[60]
		MTT/HCT117	29.95 μM (IC_50_)	Doxorubicin 0.20 μM (IC_50_)	[70]
		MTT/CCRF-CEM	32.26 μM (IC_50_)	Doxorubicin 0.003 μM (IC_50_)	[70]
		MTT/K562	9.1 μM (IC_50_)	Doxorubicin 0.04 μM (IC_50_)	[70]
		MTT/HL60	23.73 μM (IC_50_)	Doxorubicin < 0.001 μM (IC_50_)	[70]
	Antiviral	CPE inhibition/H1N1	45.5 μM (IC_50_)	Osehamivir 0.025 μM (IC_50_)	[56]
Carabrol-4-O-palmitate (**58**)	Cytotoxicity	MTA/HL60	45.85 μM (IC_50_)	Cisplatin 2.32 μM (IC_50_)	[53]
Dicarabrol (**60**)	Cytotoxicity	CCK-8/K562	1.2 μM (IC_50_)	Taxol 3.8 μM (IC_50_)	[56]
		CCK-8/MCF-7	3.3 μM (IC_50_)	Taxol 2.3 μM (IC_50_)	[56]
		CCK-8/HeLa	0.61 μM (IC_50_)	Taxol 2.9 μM (IC_50_)	[56]
		CCK-8/DU145	0.31 μM (IC_50_)	Taxol 3.1 μM (IC_50_)	[56]
		CCK-8/U937	0.15 μM (IC_50_)	Taxol 2.1 μM (IC_50_)	[56]
		CCK-8/H1975	1.4 μM (IC_50_)	Taxol 2.8 μM (IC_50_)	[56]
		CCK-8/SGC-7901	0.71 μM (IC_50_)	Taxol 9.8 μM (IC_50_)	[56]
		CCK-8/A549	2.7 μM (IC_50_)	Taxol 2.1 μM (IC_50_)	[56]
		CCK-8/MOLT-4	1.3 μM (IC_50_)	Taxol 2.7 μM (IC_50_)	[56]
		CCK-8/HL60	0.10 μM (IC_50_)	Taxol 3.8 μM (IC_50_)	[56]
	Antiviral	CPE inhibition/H1N1	15.9 μM (IC_50_)	Osehamivir 0.025 μM (IC_50_)	[56]
		CPE inhibition/H3N2	30.0 μM (IC_50_)	Osehamivir 0.015 μM (IC_50_)	[56]
	Antimycobacterial	GFPMA/*Mycobacterium tuberculosis* strains H37Rv, H37Ra	3.7 μM (MIC)	Isoniazid, 2.0 μM (MIC)	[56]
Dicarabrol A (**61**)	Cytotoxicity	MTT/HL60	8.7 μM (IC_50_)	Doxorubicin	[63]
Dicarabrol B (**62**)	Cytotoxicity	MTT/HL60	20.0 μM (IC_50_)	Doxorubicin	[62]
		MTT/A549	20.0 μM (IC_50_))	Doxorubicin	[62]
Dicarabrol C (**63**)	Cytotoxicity	MTT/HL60	3.6 μM (IC_50_)	Doxorubicin	[62]
Dicarabrone A (**64**)	Cytotoxicity	MTT/HL60	9.1 μM (IC_50_)	Doxorubicin	[64]
Dicarabrone B (**65**)	Cytotoxicity	MTT/HL60	8.2 μM (IC_50_)	Doxorubicin	[64]
Dicarabrone C (**66**)	Cytotoxicity	MTT/HL60	8.2 μM (IC_50_)	Doxorubicin	[63]
Carabrodilactone A (**67**)	Cytotoxicity	MTT/A549	4.34 μM (IC_50_)	Doxorubicin 0.05 μM (IC_50_)	[65]
		MTT/HCT117	3.08 μM (IC_50_)	Doxorubicin 0.05 μM (IC_50_)	[65]
		MTT/MDA-MB 231	8.05 μM (IC_50_)	Doxorubicin 0.05 μM (IC_50_)	[65]
		MTT/BEL 7404	3.2 μM (IC_50_)	Doxorubicin 0.05 μM (IC_50_)	[65]
Carpedilactone A (**72**)	Cytotoxicity	MTT/A549	2.63 μM (IC_50_)	Doxorubicin 0.05 μM (IC_50_)	[71]
		MTT/BEL 7404	5.53 μM (IC_50_)	Doxorubicin 0.22 μM (IC_50_)	[71]
		MTT/HLF	1.45 μM (IC_50_)	Doxorubicin 0.07 μM (IC_50_)	[71]
		MTT/CCRF-CEM	0.14 μM (IC_50_)	Doxorubicin 0.01 μM (IC_50_)	[71]
Carpedilactone B (**73**)	Cytotoxicity	MTT/A549	9.53 μM (IC_50_)	Doxorubicin 0.05 μM (IC_50_)	[71]
		MTT/BEL 7404	14.19 μM (IC_50_)	Doxorubicin 0.22 μM (IC_50_)	[71]
		MTT/HLF	3.31 μM (IC_50_)	Doxorubicin 0.07 μM (IC_50_)	[71]
		MTT/CCRF-CEM	0.32 μM (IC_50_)	Doxorubicin 0.01 μM (IC_50_)	[71]
Dipulchellin A (**74**)	Cytotoxicity	MTT/HL60	8.9 μM (IC_50_)	Doxorubicin	[63]
Faberidilactone A (**75**)	Cytotoxicity	MTT/HCT117	3.44 μM (IC_50_)	Doxorubicin 0.20 μM (IC_50_)	[70]
		MTT/CCRF-CEM	2.71 μM (IC_50_)	Doxorubicin 0.003 μM (IC_50_)	[70]
		MTT/K562	2.63 μM (IC_50_)	Doxorubicin 0.04 μM (IC_50_)	[70]
		MTT/HL60	4.95 μM (IC_50_)	Doxorubicin < 0.001 μM (IC_50_)	[70]
Faberidilactone C (**76**)	Cytotoxicity	MTT/HCT117	4.00 μM (IC_50_)	Doxorubicin 0.20 μM (IC_50_)	[70]
		MTT/CCRF-CEM	2.66 μM (IC_50_)	Doxorubicin 0.003 μM (IC_50_)	[70]
		MTT/K562	4.92 μM (IC_50_)	Doxorubicin 0.04 μM (IC_50_)	[70]
		MTT/HL60	8.12 μM (IC_50_)	Doxorubicin < 0.001 μM (IC_50_)	[70]

**Abbreviations**: A375: Human malignant melanoma cell line; A549: Lung adenocarcinoma epithelial cell line; BEL 7404: Human liver carcinoma cell line; Caco-2: Human colorectal adenocarcinoma cells line; Caski: Human cervical carcinoma; CCRF-CEM: Human leukemic cell line; CNE2: Human nasopharyngeal carcinoma cell; DU145: Human prostate cancer cell line; GES-1: Human gastric cell line; GP2-293:Human embryonic kidney line; H1975: Human non-small lung cancer cell line; HCC: Human hepatocellular carcinoma cell line; HCT116: Human colon cancer cell line; HCT-15: Human colon adenocarcinoma colorectal adenocarcinoma; HCT117: Colon carcinoma cell line; Hep3B: Human hepatoma cell line; HepG2: Human liver cancer cell line; HeLa: Human cervical cancer cell line; HGC-27: Human gastric cancer cell line; HL60: Human leukemia cell line; HLF: Human hepatoma cell line; HTC-15: Human colorectal carcinoma cell line; Huh7: Human hepato-cellular carcinoma cell line; ISK: Human endometrial epithelial cell line; K562: Human immortalized myelogenous leukemia cell line; L1210: Mouse lymphocytic leukemia cell line; L-O2: Normal human hepatic cell line; MCF-7: Human breast cancer cell line; MDA-MB-231: Human breast cancer cell line; MOLT-4: Human T lymphoblast cell line; MDCK: Madin-Darby canine kidney; NCM460: Human normal colonic epithelial cell line; RBE: Human esophageal cancer cell line; SGC-7901: Human gastric cancer cell line; SH-SY5Y: Human neuroblastoma cell line; SK-MEL-2: Human melanoma cell line; SK-OV-3: Human ovarian cancer cell line; SMMC-7721: Human hepatocarcinoma cell line; SW620, SW480: Human colorectal cancer cell line; U937: Human leukemia cell line; XF-498: Human central nervous system solid tumor cell line; CCK-8: Cell counting Kit-8; CPE: Cytopathic effect; EC50: Half maximal effective concentration; H3N2: Influenza A Virus subtype H3N2; H1N1: Influenza A Virus subtype H1N1; LPS: lipopolysaccharide; MTT: 3-(4,5-Dimethylthiazol-2-yl)-2,5-diphenyltetrazolium bromide; MIC: Minimum inhibitory concentration; NO: Nitric oxide; GFPMA: Green fluorescent protein microplate assay; SRB: Sulforhodamine B; MTS: (3-(4,5-Dimethylthiazol-2-yl)-5-(3-carboxymethoxyphenyl)-2-(4-sulfophenyl)-2H-tetrazoliuminner salt).

## Data Availability

Not applicable.

## Abbreviations

ABTS	2,2′-Azinobis-3-ethylbenzothiazoline-6-sulfonic acid
C452	Cysteine 452
CAT	Catalase
CH_2_Cl_2_	Dichloromethane
CHCl_3_	Chloroform
COX-2	Cyclooxygenase
DEET	N,N-Diethyl-meta-toluamide
DPPH	1,1-Diphenyl-2-picrylhydrazyl
DTT	Dithiothreitol
ECD	Electronic circular dichroism
EtOH	Ethanol
EtOAc	Ethyl acetate
FDI	Feeding-deterrence index
FIC	Ferrous ion chelating
GC-MS	Gas chromatography–mass spectrometry
GLUT1	Glucose transporter-1
Gpx	Glutathione peroxidase
GSH	Glutathione
HIF-1α	Hypoxia-inducible factor-1α
HINAE	Hirame natural embryo
HK2	Hexokinase-2
HPLC-ESI-Q-TOF-MS/MS	Electrospray ionization-quadrupole time-of-flight mass spectrometry
HSCCC	Highspeed counter-current chromatography
IC_50_	Half-maximal inhibitory concentration
IL-6	Interleukin-6
IL-4	Interleukin-4
IL-13	Interleukin-13
iNOS	Inducible nitric oxide synthase
IR	Infrared
LD_50_	Half maximal lethal concentration
LDHA	Lactate dehydrogenase A
MDA	Malondialdehyde
MeOH	Methanol
MyD-88	Myeloid-differential-factor-88
NMR	Nuclear magnetic resonance
NOS	Nitric oxide scavenging
NQO1	NAD(P)H:quinone oxidoreductase
Nrf2	NF-E2-related factor 2
PARP	Poly(ADPribose) polymerase
PE	Petroleum ether
PGE_2_	Prostaglandin E_2_
PKM2	Pyruvate kinase M2
RP-18	Reversed phase-18
SiO_2_ CC	Silica gel column chromatography
SOD	Superoxide dismutase
TDDFT-ECD	Time-dependent density-functional-theory electronic circular dichroism
TLC	Thin layer chromatography
TLRs	Toll-like receptors
TRIF	Toll-interleukin-1 receptor domain-containing adapter inducing interferon-β
